# LncRNA SLC7A11AR promotes lung adenocarcinoma progression by inhibiting ferroptosis via promoting SLC7A11 expression

**DOI:** 10.7150/ijbs.112233

**Published:** 2025-07-11

**Authors:** Haoqing Zhai, Xudong Xiang, Jun Pu, Xiaoqun Niu, Jie Gao, Dengcai Mu, Jia Du, Yao Li, Laihao Qu, Baiyang Liu, Yongbin Chen, Cuiping Yang

**Affiliations:** 1 State Key Laboratory of Genetic Evolution & Animal Models, the Key Laboratory of Animal Models & Human Disease Mechanisms of Yunnan Province, Kunming Institute of Zoology, Chinese Academy of Sciences, 650201, Kunming, Yunnan, China.; 2 Kunming College of Life Science, University of Chinese Academy of Sciences, Beijing, 100049, China.; 3 Kunming Medical University, Kunming, Yunnan 650118, China.; 4 The First Affiliated Hospital of Zhengzhou University, Zhengzhou 450052, China.; 5 The International Peace Maternity and Child Health Hospital, School of Medicine, Shanghai Jiao Tong University, Shanghai, China.; 6 Shanghai Key Laboratory of Embryo Original Diseases, Shanghai 200030, China.

**Keywords:** SLC7A11AR, miR-150-5p, SLC7A11, ferroptosis, lung adenocarcinoma (LUAD)

## Abstract

Non-small cell lung cancer (NSCLC) is a prevalent classification of human lung cancer with a variety of clinical pathological features. Several key factors and associated signaling pathways have played pivotal roles in the progression of NSCLC and serve as potential therapeutic targets. However, the therapeutic efficacy is still limited, and novel biomarkers and key regulators are inevitable. We found a human-specific long non-coding RNA (lncRNA, ENST00000504300) induced by the inflammatory pathway, termed SLC7A11AR (SLC7A11 associated lncRNA), which was highly expressed in lung adenocarcinoma (LUAD) cell lines but not in lung squamous cell carcinoma (LUSC). Our research showed that higher SLC7A11AR expression correlates with a poorer clinical prognosis. Depleting SLC7A11AR restrains tumor cell proliferation, migration, and xenograft tumor formation by promoting ferroptosis. Bioinformatic analysis and dual luciferase reporter assays revealed that SLC7A11AR binds directly to miR-150-5p, weakening the inhibition on its downstream target SLC7A11, a key ferroptosis inhibitor in NSCLC. In cancerous tissues, SLC7A11AR was upregulated, while miR-150-5p was downregulated compared to control tissues. Enforced miR-150-5p expression inhibited tumor growth. Moreover, ASOs against SLC7A11AR alone or with a ferroptosis agonist significantly suppressed tumor progression. Our results suggest that the SLC7A11AR/miR-150-5p/SLC7A11 axis plays an oncogenic role in LUAD development and has the potential to be novel therapeutic targets, presenting new opportunities for LUAD treatment in the future.

## Introduction

Lung cancer, which initiates within the bronchial tubes or lung glands, ranks among the most prevalent cancers globally, both in terms of its occurrence frequency and fatality rate, accounting for 12.4% and 18.7%, respectively^1^. Lung cancer is mainly categorized into NSCLC (non-small cell lung cancer) and SCLC (small cell lung cancer). NSCLC, which represents the majority of cases, is further divided into LUAD (lung adenocarcinoma), LUSC (squamous cell carcinoma), and large cell carcinoma^2^. Even though significant advancements have been made in treatment options, including targeted therapies such as EGFR inhibitors and immune checkpoint inhibitors, the overall five-year survival rate for lung cancer remains below 20%^3^. This disappointing statistic can be partially due to a limited understanding of how NSCLC develops and a deficiency in early diagnostic modalities and efficacious therapeutic targets. Therefore, it is still urgent to verify novel biomarkers critical for earlier detection and the development of more effective lung cancer treatment strategies.

Genetic and epigenetic modifications are globally acknowledged as crucial reasons for cancer progression^4^. Recent transcriptome analyses reveal that about 75% of the human genome is transcribed into RNAs. However, around 3% of the genome is involved in protein coding, and the remaining portion consists of non-coding RNAs (ncRNAs)^5^. Long non-coding RNAs (lncRNAs), defined as linear ncRNAs of 200 or more nucleotides, have attracted considerable attention for their role in cancer development^6^, ^7^. Growing evidence suggests that dysregulated lncRNA expression contributes to cancer malignant progression through diverse transcriptional, post-transcriptional, and epigenetic regulatory mechanisms^8^. LncRNAs typically possess low or negligible translational capacity.

In contrast to miRNAs that generally function in promoting mRNA degradation or modulating translation, lncRNAs influence target gene expression via diverse mechanisms that are capable of directly binding to mRNA, DNA, or proteins, thereby modulating chromatin structure, as well as governing the processes of transcription, splicing, and translation^9^. This regulatory capability enables lncRNAs to affect various procedures, including cell proliferation, differentiation, stem cell pluripotency, and multiple-therapeutic resistance^10^, ^11^. Although most lncRNAs prefer to be in the nucleus, a subset exhibit activity in the cytoplasm. These cytoplasmic lncRNAs can be secreted and transported to neighboring cells or into the serum via exosome-mediated trafficking pathways^12^. Given these properties, lncRNAs show great potential as therapeutic targets, diagnostic indicators, and malignant predictors, with lung cancer being a prominent example.

In the wake of the continuous progress in science and technology, the concept of programmed cell death (PCD) has come to the fore, explaining this phenomenon of "cell suicide" and its involvement in the progression of numerous diseases, including human cancers^13^. PCD includes various mechanisms, such as apoptosis, autophagy, and programmed necrosis, which contrasts with accidental cell death known as necrosis^14^. Programmed necrosis encompasses different cell death types, including pyroptosis, necroptosis, and ferroptosis^15^. Ferroptosis, a kind of programmed necrosis, is marked by phospholipid peroxidation and free iron-mediated Fenton reactions, distinguishing it as an iron-dependent cell death mechanism crucial to tumor initiation and progression, particularly in NSCLC^16^, ^17^. Ferroptosis can be both triggered and restrained depending on the context. In ferroptosis, the System xc- composed of the functional subunit SLC7A11 (solute carrier family 7 member 11) and the regulatory subunit SLC3A2 (solute carrier family 3 member 2), facilitates the import of cystine into cells, with glutamate exported during the process^18^. The synthesis of glutathione (GSH), a significant endogenous antioxidant, is contingent upon glutamate-cysteine ligase (GCL). When System xc- is inhibited, GSH synthesis decreases, leading to increased levels of lipid peroxides and subsequently promoting ferroptosis^19^, ^20^. Numerous studies have established that the process of ferroptosis can be instigated through the inhibition of System xc- by particular compounds, namely erastin, sulfasalazine, sorafenib, and lanperisone^21^. Concurrently, genes involved in the modulation of the catalytic subunit within System xc- exert an impact on the transcriptional or translational activities of subunits such as SLC7A11 and SLC3A2, thereby impacting the biological processes associated with ferroptosis^22^, ^23^. Thus, understanding the roles of System xc-, SLC7A11, and various regulatory pathways may pave the way for new therapeutic strategies to combat NSCLC.

Inflammation can be a double-edged sword. When its regulation and occurrence are short-term, inflammation functions through a protective mechanism. However, if inflammation becomes unregulated, it has the potential to lead to a diverse range of diseases, with lung cancer and other chronic ailments being of particular concern^24^, ^25^. Reactive oxygen species (ROS) produced in reaction to injuries or infections possess the capacity to activate epithelial cells, causing them to release more ROS and pro-inflammatory cytokines. These cytokines, including TGF-β, TNF-α, and IL-6, bind to their corresponding receptors and trigger inflammation pathways, which can either lead to the resolution of inflammation or result in its perpetuation, facilitating the development of chronic injury or the formation of tumors within the lungs^26^. Oncogenic proteins like STAT can be activated during cytokine storms, promoting cell proliferation and inhibiting apoptosis, which can contribute to lung cancer progression^27^. Targeting inflammation might be a promising therapeutic approach for cancers. However, several challenges persistently impede the progression of anti-inflammatory and anti-cancer therapies, including but not limited to diminished treatment effectiveness, suboptimal drug distribution within the body, and the occurrence of adverse side effects. Such obstacles continue to pose significant barriers to the normal development and implementation of more potent and targeted treatment modalities in anti-inflammation and anti-cancer. In this study, an inflammatory signaling-induced upregulation of lncRNA-SLC7A11AR in lung adenocarcinoma caused an unfavorable clinical prognosis. Therefore, we decided to elucidate the underlying mechanism through which SLC7A11AR facilitates the progression of LUAD.

## Materials and Methods

### Cell culture, constructs, transfection and lentiviral infection

The BEAS-2B cell line and its relevant culture medium were from the Cell Bank of Kunming Institute of Zoology, Chinese Academy of Sciences. The HEK-293T cell line was from ATCC. Regarding the lung cancer cell lines, H358, H1650, H1975, H1299, SPC-A1, HCC827, H1299, and A549 were obtained from Cobioer within the territory of China. PC9, SK-MES-1, and H226 were from Procell, and all cell lines have STR profiles. The designated cancer cell lines were cultivated in RPMI-1640 medium (Gibco), augmented with 1% penicillin/streptomycin and 10% fetal bovine serum (FBS). Meanwhile, HEK-293T cells used a DMEM medium (Gibco).

The SLC7A11AR obtained via RT-PCR subcloned into either the pCDH-MSCV-E2F-eGFP lentiviral vector or the pCDNA3.1 vector, and a 6×Myc tag was addicted to the C-terminus of SLC7A11AR during this process. Meanwhile, the SLC7A11 gene constructed into the pCDH-MSCV-E2F-eGFP lentiviral vector has a 3×Flag tag at its C-terminus. Furthermore, we synthesized shRNA targeting SLC7A11AR and subcloned it into the lentiviral vector pLKO.1 (Addgene, Cambridge, USA). Lentivirus production such as control shRNA, SLC7A11AR-specific repressing shRNA, pCDH-Vec, and pCDH-SLC7A11AR were transfected into HEK-293T cells using calcium phosphate methods with pMD2.G/psPAX2 plasmids. At 48-72 hours after cell transfection, the supernatants of the cells were harvested and subsequently utilized to infect the designated cell lines, and puromycin was addicted to select stable cell lines.

Control siRNA, siRNA targeting NFKB1 and ACSL4; miR-NC (microRNA control), miR-150-5p mimics, Anti-Ctrl (microRNA inhibitor control), and miR-150-5p inhibitors; both the control ASO and the ASOs specifically designed to deplete the expression of SLC7A11AR were from Shanghai GenePharma. (China). The designated cells transfected with the products via Lipofectamine 2000 (Invitrogen) were harvested for the following experiments. The oligonucleotide sequences employed in this research are presented in Table S2.

### Cell proliferation, colony formation, cell migration and cell flow cytometry assays

Growth curves, colony formation, and wound healing experiments were carried out according to the previously established protocols^28^. In brief, about the cell proliferation assay, the designated cells were seeded (6×10³ cells per well) in a 12-well plate (NEST, China, Cat.712001) and counted using an automated cell counter (Countstar, Shanghai Ruiyu Biotechnology Co., Ltd., China, IC1000) at the same time each day. For the colony formation assay, the indicated cells plated (400 cells per well) in a 6-well plate (NEST, China, Cat.703001) were immobilized in 4% PFA after reaching the appropriate colony size. Subsequently, they were stained with 0.5% crystal violet to facilitate imaging and cell counting procedures. In the wound healing assay, the designated cells were seeded in a 6-well plate, scratched a linear wound using a pipette tip after complete adherence, and washed out the detached cells with PBS. Subsequently, images documenting the wound healing progression were acquired with designated time intervals by a Nikon inverted microscope (Ti-S). The wound healing area was quantified using ImageJ. In the context of the trans-well migration assay, a volume of 100 µL of serum-free medium with 2×10^4^ cells contained in the upper compartment of a 24-well trans-well plate (Corning Life Sciences, Cat.3422), concurrently, the lower chamber containing 600 µL of medium with 10% FBS was immobilized using 4% PFA. after 24-hour incubation. Cells were thoroughly washed and then subjected to staining with 0.5% crystal violet. Enumerated cells presented the migration efficiency. In the process of cell cycle analysis, the cells were first subjected to digestion and subsequently washed twice with PBS. Subsequently, the cells were fixed in 75% ethanol at 4°C overnight. After completion of the fixation process, the cells were washed with PBS and stained with propidium iodide (PI) at 37°C for 30 minutes under dark conditions. Finally, the cell cycle distribution was analyzed using a FAC-Saria SORP apparatus (BD, USA).

### Immunoblot and Real-time RT-PCR assays

For immunoblot, the proteins extracted by RIPA buffer containing protease inhibitors (Complete Mini, Roche) were assessed by immunoblot using indicated primary and secondary antibodies. For real-time RT-PCR, total RNA was extracted using RNAiso Plus (Takara Bio, Beijing, China, Cat. 108-95-2) according to the manufacturer's instructions. Reverse transcription employed a reverse transcription kit (Vazyme Biotech Co., Ltd. Nanjing, China, Cat. R323-01; TIANGEN Biotech, Beijing, China, Cat. KR211-02). The Quantitative analysis utilized FastStart Universal SYBR Green Master Mix (Vazyme Biotech, Nanjing, China, Cat.Q511-02; TIANGEN Biotech, Beijing, China, Cat. FP411-02) and the Applied Biosystems 7500 instrument. In this study, the antibodies and primer sequences employed are in Table S2.

### Dual-luciferase reporter assay, Chromatin Immunoprecipitation assay (CHIP) and Immunohistochemical staining (IHC)

The upstream promoter region sequence of SLC7A11AR (-2000bp to -1bp) obtained from Ensembl (http://asia.ensembl.org/index.html) with potential for miR-150-5p binding sites within the 3'-UTR regions of SLC7A11AR and SLC7A11 were prognostically determined using the Starbase (http://starbase.sysu.edu.cn/). The wild-type and mutant DNA fragments within the promoter region of SLC7A11AR or the potential binding sequences of miR-150-5p were addicted into the pGL3-Basic luciferase reporter vector through subcloning techniques. All constructs underwent sequence verification. The indicated cells (2×10^4^ cells per well) in a 24-well plate (NEST) transfected into the respective cells with the application of Lipofectamine 2000 were lysed after 48-72 hours per the manufacturer's protocol. Subsequently, the Dual-Luciferase Kit (Promega) was employed to determine the activity levels of both firefly luciferase and Renilla luciferase. For the CHIP assay, the experimental procedure followed the manufacturer's protocol of the SimpleChIP^®^ Plus Enzymatic Chromatin IP Kit (CST, Cat.9005). 8×10^6^ cell protein extraction was collected and subjected to ChIP reactions using 2 µg of normal rabbit IgG antibody (CST, Cat.2729) and anti-NFKB1 (p50) antibody (CST, Cat.13586). The precipitated DNAs were analyzed using enrichment efficiency and fold change using the Real-Time RT-PCR. The immunohistochemical staining protocol was as previously described. Images were captured and analyzed using a microscope (Olympus BX43F, Japan), and the data was quantified (Media Cybernetics, Inc., Silver Spring, MD, USA) for further evaluation.

### Animal experiments

As the xenograft tumor growth assay described in prior studies^28^, the indicated cells (1×10^6^ cells/point) were subcutaneously implanted into male nude mice aged 4-6 weeks. The body weights and the tumor volumes were measured at regular intervals using calipers with the formula (L×W²)/2. In the end, the mice were humanely euthanized. Subsequently, the tumors were excised and weighed. In the miR-150-5p agomir treatment experiment, once the tumor volume had attained a size of 50 mm³, the mice were randomly allocated into two distinct groups, receiving miR-NC (5 nmol) and miR-150-5p mimics (5 nmol) injections around the tumor twice a week. For the ASO treatment experiment, upon tumors reaching 50 mm³, the mice were randomly allocated into six groups, receiving indicated ASOs (5 nmol) injected around the tumor twice a week via intraperitoneal injection every other day. For the lung metastasis assay, the established pLVX-Luc2-P2A-AcGFP1-puro-labeled control cell line and the SLC7A11AR knockdown cell line were injected into the mice tail vein (3×10⁶ cells per mouse). At 40 days post-injection, *in vivo* imaging and lung collection were performed. All mice were randomly assigned to groups before the transplantation of tumor cells, and the measurements of the transplanted tumors were conducted by personnel unaware of the group assignments. All experimental animals were maintained under SPF conditions, and the experimental procedures were reviewed and endorsed by the Animal Protection and Use Committee at the Kunming Institute of Zoology, Chinese Academy of Sciences.

### Ferroptosis signaling pathway examination

Transmission electron microscopy (TEM) examined the characteristic morphological traits associated with mitochondrial ferroptosis. In brief, following the digestion process and subsequent washing with PBS, the cells were subjected to fixation in 2.5% glutaraldehyde overnight. After washing, fixation, dehydration, resin embedding, polymerization, sectioning, and staining conducted in the Instrument Center of the Kunming Institute of Zoology, Chinese Academy of Sciences, the samples were for observation and photography by a transmission electron microscope (JEM-1400 Plus 80kV). To evaluate the levels of lipid peroxidation and reactive oxygen species (ROS) the designated cells were first digested, washed with PBS, and incubated with either the BODIPY 581/591 C11 lipid oxidation probe (Invitrogen, USA, D3861, 5µM) or the CM-H2DCFDA fluorescent probe (Invitrogen, USA, C6827, 5µM) for 30 minutes, followed by three washes with PBS before analyzing using a flow cytometer. Glutathione (GSH) (Reduced Glutathione Colorimetric Assay Kit, Cat. E-BC-K030-M), malondialdehyde (MDA) (Malondialdehyde Colorimetric Assay Kit, Cat. E-BC-K028-M), and ferrous ion (Fe²⁺) (Cell Ferrous Iron Colorimetric, Cat. E-BC-K881-M) level validations were according to the manual provided by Elabscience. The obtained lysates reacted with reagents provided in the kit, with absorbance measured at 405 nm, 532 nm, and 593 nm, respectively, using a microplate reader.

### ALT and AST activity assay

The activities of ALT (ALT activity detection kit, Solarbio, BC1550) and AST (AST activity detection kit, Solarbio, BC1560) were measured to evaluate whether the combined treatment of ASOs and IKE induced hepatotoxicity. The assays were strictly conducted according to the manufacturer's instructions.

### Bioinformatics analysis

Statistical analyses employed GraphPad Software 9. The expression analysis of lncRNAs and microRNAs used public data sets. Survival curves for prognostic evaluation were using the Kaplan-Meier approach. KEGG pathway enrichment profiles were analyzed using GSEA software. The specificity and sensitivity of SLC7A11AR and miR-150-5p assessment via receiver operating characteristic (ROC) curves and the area under the curve (AUC) were by the pROC R software package. Pearson's analysis was for the correlation analysis. Student's t-test was ascertained between the two experimental groups, while one-way ANOVA was among multiple-group comparisons. *P* < 0.05 (*), *P* < 0.01 (**), and* P* < 0.001 (***) were significant.

## Results

### Inflammation induced upregulation of SLC7A11AR in lung adenocarcinoma

**Figure 1:**
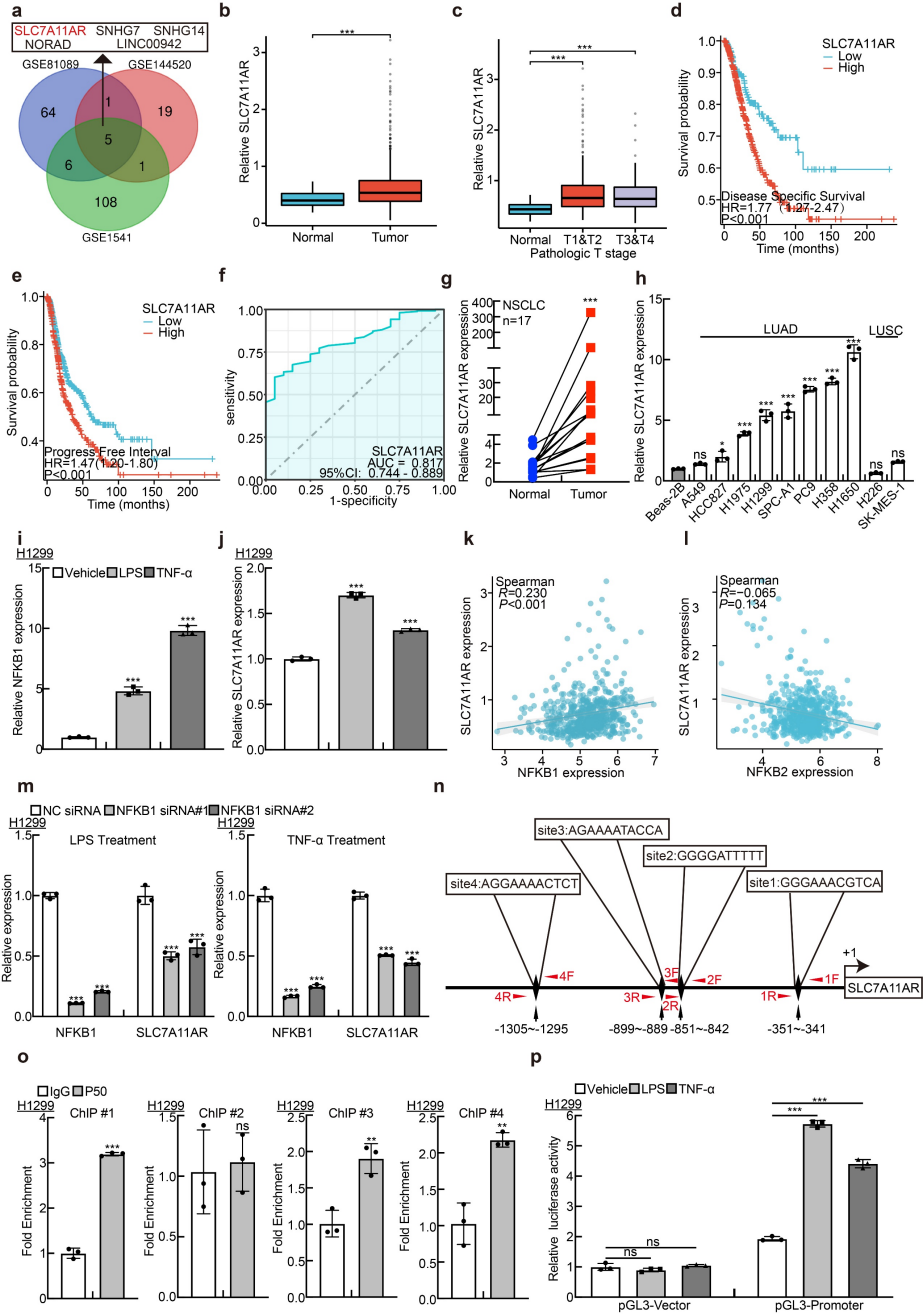
** Inflammatory induction of lncRNA SLC7A11AR highly expressed in LUAD. a** LncRNA SLC7A11AR was identified by integrative analysis using GEO datasets, GSE81089 (Blue): Next Generation Sequencing (RNAseq) from NSCLC; GSE144520 (Red): whole-transcriptome sequencing of A549 cells and cisplatin-resistant A549/DPP cells; GSE1541 (Green): whole-transcriptome sequencing of A549 cells and A549 treated with inflammatory factors (LPS and TNF-α). **b** Relative expression levels of SLC7A11AR in TCGA-LUAD (Normal: 59 cases; Tumor: 539 cases). **c** Relative expression levels of SLC7A11AR in different clinical stages of TCGA-LUAD (Normal: 59 cases; T1&T2: 468 cases; T3&T4: 68 cases). **d-e** High expression of SLC7A11AR correlates with poorer clinical prognosis, evidenced by worse DSS (Disease-Specific Survival) (**d**) and PFI (Progress Free Interval) (**e**). **f** ROC curve analysis of SLC7A11AR in LUAD uncovered AUC=0.817 using the TCGA dataset. **g** The expression of SLC7A11AR was detected in paired clinical tissue samples from NSCLC patients by Real-time RT-PCR (n=17). **h** Relative expression of SLC7A11AR in non-small cell lung cancer cell lines (A549, HCC827, H1975, H1299, SPC-A1, PC9, H358, H1650, H226, and SK-MES-1), compared to the normal bronchial epithelial cell line BEAS-2B, assessed by Real-time RT-PCR. **i-j** Using NFKB1 (**i**) as a positive control, the changes in SLC7A11AR (**j**) expression in H1299 cells treated with lipopolysaccharide (LPS) and tumor necrosis factor (TNF-α) were determined by Real-time RT-PCR. **k-l** Correlation analysis of SLC7A11AR with NFKB1 (p50) (**k**) and NFKB2(p52) (**l**) in the TCGA-LUAD dataset. **m** The expression of SLC7A11AR in H1299 cells after the knockdown of NFKB1, under conditions treated with LPS and TNF-α, was measured by Real-time RT-PCR. **n** NFKB1 binding sites on the SLC7A11AR promoter region were predicted using the JASPAR website and illustrated in the schematic diagram. **o** ChIP assay of NFKB1 enrichment at the SLC7A11AR promoter region was performed, sites #1, #2, #4, but not site #3, exhibited NFKB1 binding enrichment. **p** Luciferase reporter assays were performed under indicated conditions induced by LPS and TNF-α. * *P* < 0.05, ** P < 0.01, *** P < 0.001.

For determining the latent oncogenic lncRNAs that are involved in the advancement of NSCLC triggered by inflammatory signaling pathways, we collected and compared the lncRNA expression profiles in NSCLC tissues using different websites available datasets, including GSE81089, GSE144520, and GSE1541. The expression levels of five lncRNAs, including SLC7A11AR, SNHG7, NORAD, LINC00942, and SNHG14, were significantly elevated (Fig. 1a, Table S1). However, except for SLC7A11AR, the crucial functions of the remaining four lncRNAs have been comprehensively elucidated in lung cancer^29^-^32^. To gain a deeper insight into the role of SLC7A11AR in lung cancer, we confirmed its high expression in LUAD and its significant correlation with tumor staging. Moreover, patients with a higher expression of SLC7A11AR tended to exhibit a poorer clinical prognosis (Fig. 1b-e). To assess the clinical significance and diagnostic value of SLC7A11AR in LUAD, we applied ROC curve analysis and obtained an AUC value of 0.817 in LUAD, indicating that SLC7A11AR has the potential to function as a prognostic biomarker in LUAD (Fig. 1f). We quantified the expression level of SLC7A11AR in LUAD cancerous tissues via Real-Time RT-PCR. In contrast to the paracancerous (adjacent tumor tissue) group, a notably elevated expression of SLC7A11AR was detected in the paired LUAD cancerous tissues (Fig. 1g). In addition, our observations revealed a significant upregulation of SLC7A11AR in multiple LUAD cell lines, namely H1975, H1299, SPC-A1, PC9, H358, and H1650, excluded the LUSC cell lines, H226 and SK-MES-1, when compared with the human bronchial epithelial cell line BEAS-2B (Fig. 1h). Therefore, we decided to conduct a more in-depth characterization of the functional role played by SLC7A11AR in LUAD. Consistently, we detected that SLC7A11AR was increased upon lipopolysaccharide (LPS) and tumor necrosis factor (TNF-α) treatment in LUAD but not LUSC cell lines, indicating that SLC7A11AR was indeed induced by inflammation signaling in LUAD (Fig. 1i-j and Fig. S1a-h). To validate this finding, we then analyzed the correlation between SLC7A11AR and the key activators involved in the NF-κB signaling pathway, we analyzed key DNA-binding proteins NFKB1(p50) and NFKB2(p52) in both the canonical and non-canonical NF-κB signaling pathways^33^. Notably, NFKB1(p50) but not NFKB2(p52), were positively associated with SLC7A11AR expression (Fig. 1k-l). Furthermore, we revealed that inhibition of NFKB1, resulted in a significant decrease of SLC7A11AR, suggesting that NFKB1 directly mediated the upregulation of SLC7A11AR in LUAD (Fig. 1m). Using the JASPAR database, we predicted four NFKB1 binding sites within the promoter region of SLC7A11AR, and 3 out of 4 were subsequently validated by ChIP (Chromatin Immunoprecipitation assay) and Dual-luciferase reporter assays (Fig. 1n-p, Table S2). These results indicated that inflammation-related NF-κB signaling induces SLC7A11AR expression in LUAD. In addition, we analyzed the relationship between the gene mutation profiles (including mutations in EGFR, KRAS, or TP53) and the expression pattern of SLC7A11AR. We showed that EGFR mutation slightly increased, while KRAS mutation mildly reduced SLC7A11AR expression. However, TP53 mutation does not correlate with SLC7A11AR expression (Fig. S1i-k).

### SLC7A11AR promotes tumor cell proliferation and migration

**Figure 2:**
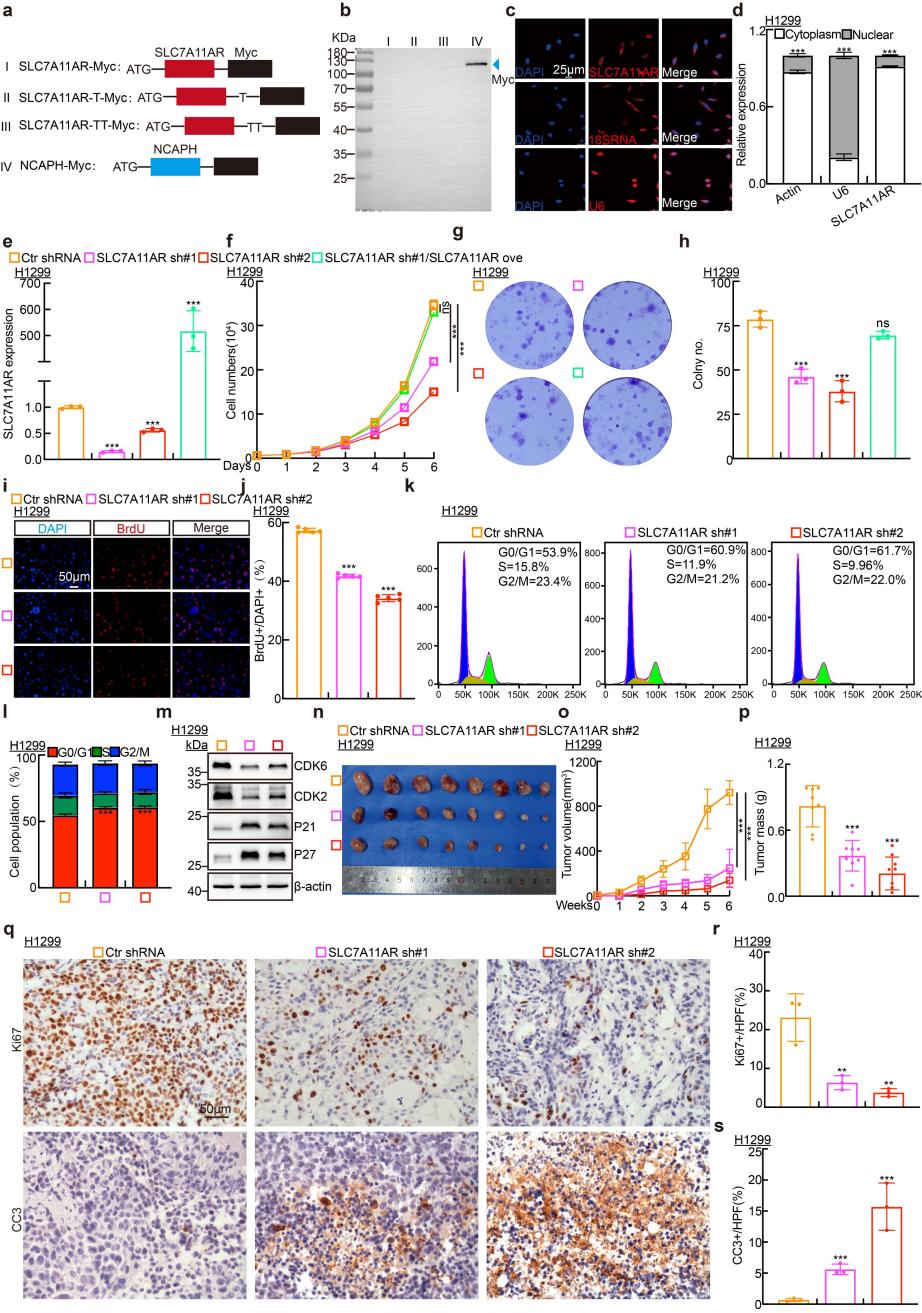
** SLC7A11AR promotes LUAD tumor cell proliferation. a** Schematic picture of SLC7A11AR-Myc tagged constructs. **b** Immunoblot to detect whether SLC7A11AR can code for proteins or short peptides. NCAPH-Myc protein was used as a control (blue arrow). **c-d** Fluorescence in situ hybridization (FISH) (**c**) and nuclear-cytoplasmic separation experiment (cell fractionation) (**d**) to detect the localization of SLC7A11AR. Scale bar = 25μm. **e** Efficiency of SLC7A11AR knockdown and overexpression in H1299 cells as assessed by Real-time RT-PCR. **f-h** Knockdown of SLC7A11AR significantly inhibits the proliferation (**f**) and colony formation ability (**g-h**) of H1299 cells. (**h**) is the statistical result for (**g**). **i-j** The BrdU incorporation assay in H1299 cells. (**j**) is the statistical result for (**i**), the experiment was repeated three times. Scale bar=50μm.** k-l** PI staining and flow cytometry to assess the impact of SLC7A11AR knockdown on the G0/G1 cell cycle transition in H1299 cells, (**l**) is the quantitative result for (**k**). (**m**) Immunoblots to detect changes in expressions of key cell cycle regulators, including CDK2, CDK6, P21, and P27 in indicated cells, β-actin was used as an internal control. (**n-p**) SLC7A11AR knockdown repressed xenograft tumor growth *in vivo*, tumor images (**n**), tumor volumes (**o**), and tumor weights (**p**) were shown. (**q-s**) Results of immunohistochemical staining (**q**) and quantification of positive signals for Ki67 (**r**) and Cleaved Caspase 3 (CC3) (**s**). Scale bar = 50μm. * *P* < 0.05, ** P < 0.01, *** P < 0.001. Ove = over-expression; sh#1 = shRNA#1; sh#2 = shRNA#2; HPF = high power field.

To verify that SLC7A11AR does not possess the ability to encode proteins or short peptides, the full-length SLC7A11AR was subcloned into the eukaryotic expression vector pcDNA3.1 following three distinct coding modalities. After transfecting these plasmids into HEK-293T cells, an immunoblot and Immunoprecipitation (IP) were performed to detect the Myc-tagged protein. Myc-tagged NCAPH was prominently detected^34^, while neither weak nor strong exposure revealed that Myc-tagged SLC7A11AR proteins were absent (Fig. 2a-b and Fig. S2a). Consistent with the online predicted result, SLC7A11AR is a classic long non-coding RNA (Fig. S2b). In addition, SLC7A11AR preferred to localize within the cytoplasm. This prediction was further corroborated by RNA fluorescence in situ hybridization (FISH) assays and nuclear-cytoplasmic fractionation experiments (Fig. 2c-d and Fig. S2c-e). To decipher the functional mechanism of SLC7A11AR, two distinct lentiviral short shRNAs were employed to effectively deplete the expression of SLC7A11AR in the H1299, H1975, and SPC-A1 cell lines, and a scramble shRNA was set up as a control (Fig. 2e and Fig. S2f-g). As expected, the knockdown of SLC7A11AR inhibited tumor cell proliferation, while forced expression of SLC7A11AR overcame this phenotype validated by growth curves and colony formation assay (Fig. 2f-h and Fig. S2h-m). Furthermore, the BrdU incorporation ability decreased upon SLC7A11AR knockdown (Fig. 2i-j and Fig. S2n-o). Subsequently, we assessed potential disparities in cell cycle transition using flow cytometry, which revealed that SLC7A11AR knockdown led to increased G0/G1 phase cell population (Fig. 2k-l and Fig. S2p). Consistently, immunoblot analysis showed that SLC7A11AR knockdown reduced CDK2 and CDK6 expression while increasing P21 and P27 levels (Fig. 2m and Fig. S2q), key regulators of the G0/G1 cell cycle checkpoint.

The xenograft tumor formation assay was performed to elucidate the *in vivo* function of SLC7A11AR. Six-week-old male nude mice were allocated into three groups, and the indicated H1299 cell lines (1x10^6^ cells/point) containing either scramble shRNA or SLC7A11AR-targeted shRNAs were subcutaneously injected. Our research demonstrated that SLC7A11AR depletion significantly inhibited tumor progression *in vivo* (Fig. 2n-p). Immunohistochemistry (IHC) assay was analyzed to detect the expressions of Ki67, a biomarker for cell proliferation, and cleaved caspase 3 (CC3), a biomarker for apoptosis, within the xenograft tumors, and the results showed that knockdown of SLC7A11AR inhibited tumor growth, while promoted tumor cell apoptosis (Fig. 2q-s).

**Figure 3:**
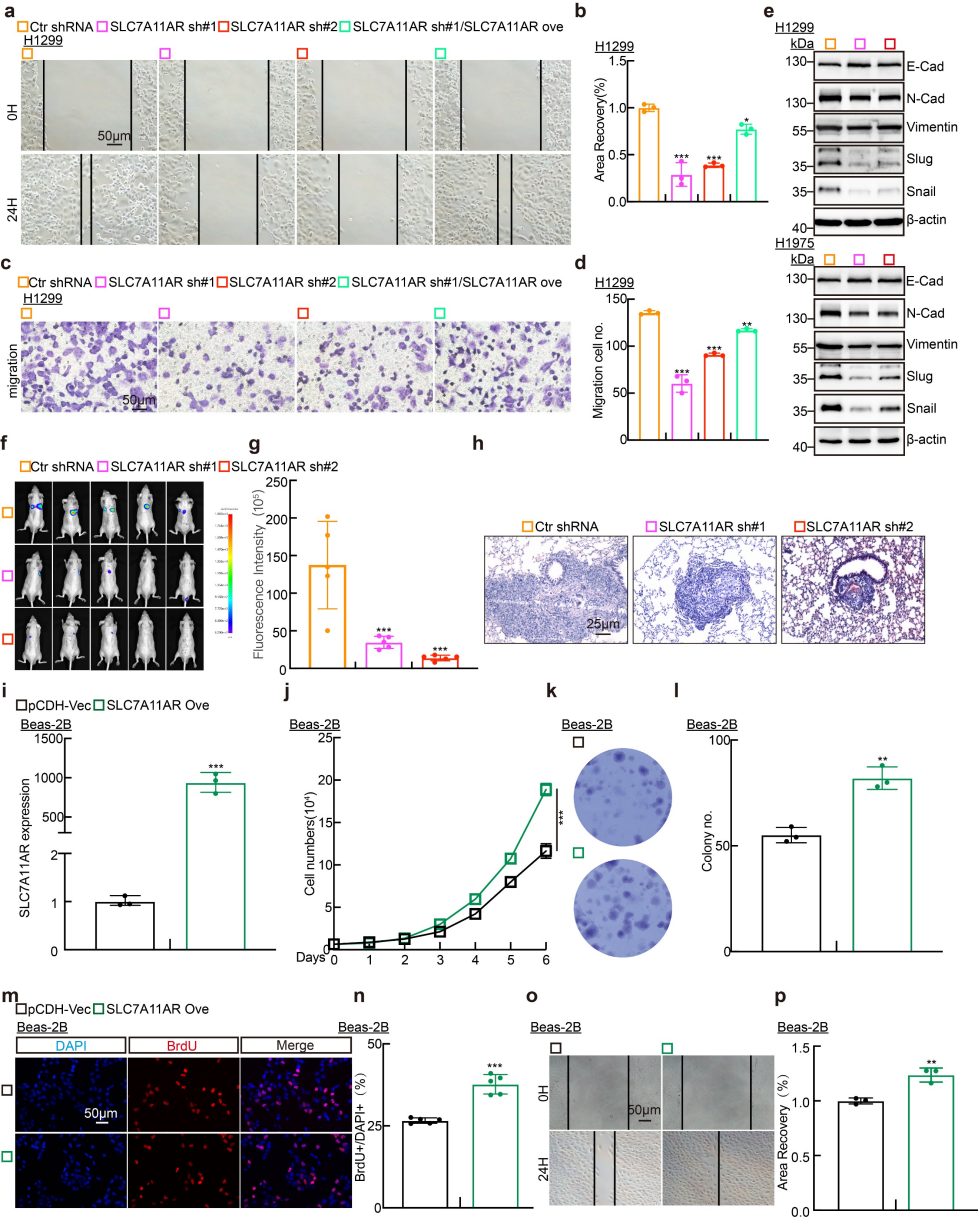
** SLC7A11AR plays as an oncogene in LUAD. a-d** Wound healing (**a-b**) and trans-well (**c-d**) assays indicating the migration ability of H1299 cells following SLC7A11AR knockdown, with quantitative statistics presented. Scale bar = 50μm. **e** Immunoblots to detect the protein expressions of the key regulators involved in cell migration, including E-cadherin, N-cadherin, Vimentin, Slug, and Snail following SLC7A11AR knockdown in indicated cells. **f** *In vivo* imaging of lung metastasis. **g** Statistical results of fluorescence quantification. **h** H&E staining results of mouse lung tissue. Scale bar=25μm. **i** The overexpression efficiency of SLC7A11AR in BEAS-2B cells. **j-p** The overexpression of SLC7A11AR promotes the tumor cell proliferation (**j**), colony formation (**k-l**), the BrdU incorporation assay (**m-n**), and migration (**o-p**) abilities of BEAS-2B cells, reciprocal statistical results were presented. Scale bar=50μm. * *P* < 0.05, ** P < 0.01, *** P < 0.001. pCDH-Vec = pCDH lentiviral plasmid vector control; Ove= over-expression.

To determine whether SLC7A11AR regulates tumor cell migration, we conducted wound healing and trans-well assays. The results demonstrated that SLC7A11AR knockdown markedly retarded tumor cell migration (Fig. 3a-d and Fig. S3a-e). Likewise, immunoblot assays investigated the gene expressions intimately associated with the epithelial-mesenchymal transition (EMT) process. The results demonstrated that the knockdown of SLC7A11AR led to a significant elevation in the expression of E-cadherin while a notable reduction in the expressions of N-cadherin, vimentin, Slug, and Snail (Fig. 3e). We evaluated the lung metastatic capacity after SLC7A11AR knockdown via tail vein injection assay. Consistent results from *in vivo* imaging, quantitative lung fluorescence analysis, and H&E staining collectively demonstrated that SLC7A11AR knockdown significantly suppressed tumor metastasis to the lungs (Fig. 3f-h). Notably, we uncovered that forced-expression of SLC7A11AR promoted the oncogenic transformation of normal Beas-2B cells, validated by the growth curve, colony formation, BrdU incorporation assays, and wound healing, supporting that SLC7A11AR functions as a crucial oncogene in LUAD (Fig. 3i-p).

### SLC7A11AR promotes SLC7A11 expression and inhibits ferroptosis

**Figure 4:**
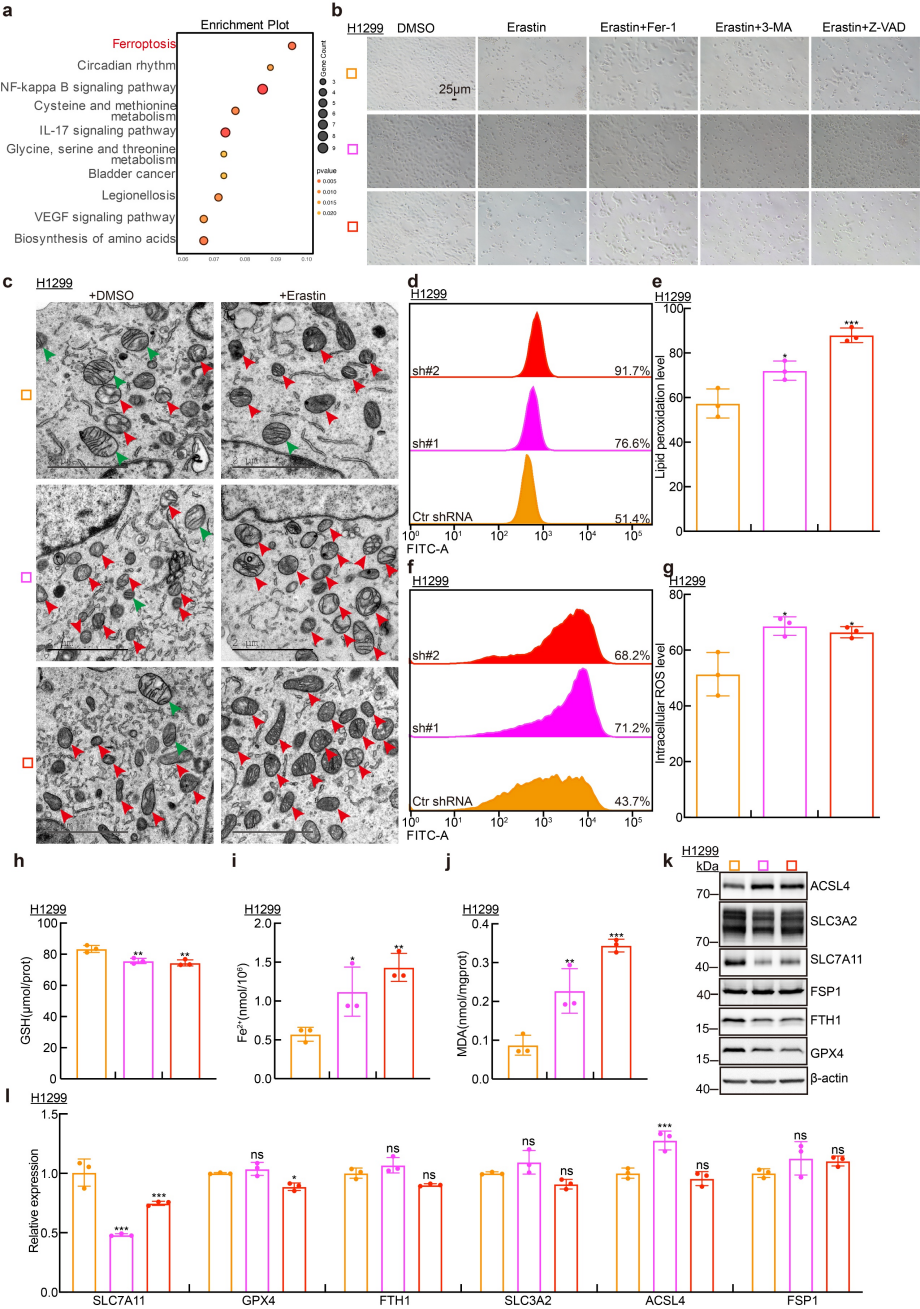
** SLC7A11AR promotes SLC7A11 expression at the transcriptional level and inhibits ferroptosis. a** Pathway enrichment analysis of the transcriptomic data upon SLC7A11AR knockdown in H1299 cells.** b** Morphological changes in cells after SLC7A11AR knockdown and treatment with ferroptosis inducers (erastin), ferroptosis inhibitors (Fer-1), autophagy inhibitors (3-MA), and apoptosis inhibitors (Z-VAD). Scale bar=25μm. **c** Transmission electron microscopy showing mitochondrial morphological changes after SLC7A11AR knockdown with or without erastin (10µM) treatment. Scale bar =2μm. **d-g** Flow cytometry detecting lipid peroxidation levels (**d-e**) and reactive oxygen species (ROS) levels (**f-g**) in H1299 cells after SLC7A11AR depletion, with (**e**) and (**g**) showing the statistical results. **h-j** Changes in glutathione (GSH) (**h**), ferrous ions (Fe²⁺) (**i**), and malondialdehyde (MDA) (**j**) levels in H1299 cells after SLC7A11AR knockdown, respectively. **k-l** Immunoblots (**k**) and Real-time RT-PCRs (**l**) to detect the protein and RNA expressions of the key factors involved in ferroptosis following SLC7A11AR knockdown in indicated cells. * *P* < 0.05, ** P < 0.01, *** P < 0.001.

To uncover the underlying mechanism by which SLC7A11AR increases tumor progression, we collected RNA samples from H1299 cell lines for transcriptome sequencing. Bioinformatic analysis detected a strong association between SLC7A11AR expression and the ferroptosis signaling (Fig. 4a).

Furthermore, we found that only the ferroptosis inhibitor (Fer-1)^35^, but neither the autophagy inhibitor (3-MA) nor the apoptosis inhibitor (Z-VAD-FMK)^36^, ^37^, was able to reverse ferroptosis phenotype upon ferroptosis agonist (erastin) treatment, verified by cellular morphological examination(Fig. 4b)^38^. Consistently, transmission electron microscopy (TEM) examination revealed that SLC7A11AR knockdown caused mitochondrial deregulated characteristic of ferroptosis, including the disappearance of mitochondrial cristae, mitochondrial shrinkage, and increased membrane density (Fig. 4c). In line with the mitochondrial morphological changes, we observed a marked elevation in intracellular lipid peroxidation and the levels of reactive oxygen species (ROS), malondialdehyde (MDA) and ferrous ions (Fe²^+^) levels, while a decrease of intracellular glutathione (GSH) levels, following SLC7A11AR knockdown (Fig. 4d-j and Fig. S4a-e). The expressions of the key regulators involved in ferroptosis, including ACSL4, SLC3A2, SLC7A11, FTH1, GPX4 and FSP1, were further detected by immunoblot and Real-Time RT-PCR. Our results revealed that SLC7A11 unanimously decreased, but not SLC3A2, FTH1, GPX4 and FSP1, upon SLC7A11AR knockdown at RNA and protein levels. On the contrary, ACSL4 proteins, but not RNAs, were consistently increased after SLC7A11AR knockdown (Fig. 4k-l and Fig. S4f-g). We further knocked down ACSL4 by siRNA transfection. Growth curve and colony formation assays demonstrated that ACSL4 knockdown significantly rescued the growth inhibition caused by SLC7A11AR depletion (Fig. S4h-k). The above findings indicate that SLC7A11AR probably promotes SLC7A11 expression at the transcriptional level via competing endogenous RNA role (ceRNA) to repress ferroptosis^39^.

### SLC7A11AR is the sponge lncRNA for miR-150-5p to induce SLC7A11 expression in LUAD

**Figure 5:**
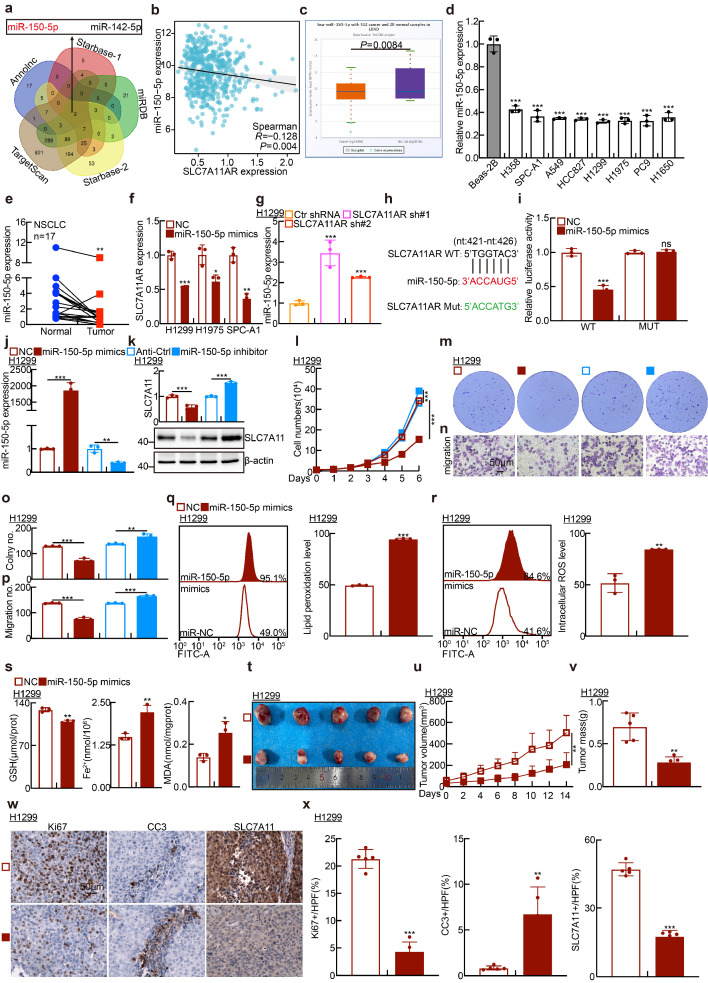
** MiR-150-5p promotes ferroptosis in lung adenocarcinoma. a** Screening potential microRNAs that may bind to both SLC7A11AR and SLC7A11 using multiple databases: Annolnc dataset (Blue); SLC7A11AR as target gene in Starbase dataset (Red); miRDB dataset (Green); SLC7A11 as target gene in Starbase dataset (Yellow); TargetScan dataset (Brown). **b** Correlation analysis of SLC7A11AR with miR-150-5p using the TCGA-LUAD dataset. **c** Relative expression levels of miR-150-5p in TCGA-LUAD (Normal: 20 cases; Tumor: 512 cases). **d** Relative expression levels of miR-150-5p in LUAD cell lines (H358, SPC-A1, A549, HCC827, H1299, H1975, PC9, H1650) compared to the normal bronchial epithelial cell line BEAS-2B, assessed by Real-time RT-PCR. **e** The expression of miR-150-5p was detected in paired clinical tissue samples from LUAD patients by Real-time RT-PCR (n=17). **f** Relative SLC7A11AR expression levels in H1299, H1975, and SPC-A1 cells transfected with miR-150-5p mimics compared with miR-NC group were evaluated by Real-time RT-PCR. **g** Relative miR-150-5p expressions after SLC7A11AR knockdown, were evaluated by Real-time RT-PCR. **h-i** Binding sites of miR-150-5p to SLC7A11AR predicted by miRDB, with the schematic of wild-type (WT) and mutant (MUT) luciferase reporter plasmids shown (**h**). HEK-293T cells co-transfected with specific plasmids and miR-NC or miR-150-5p mimics were collected to detect luciferase activity (**i**). **j** Transfection efficiency of miR-150-5p mimics and inhibitors in H1299 cells. **k** The RNA (top) and protein (bottom) expressions of SLC7A11 were detected by Real-time RT-PCR and Immunoblots, respectively. **l-p** Cell proliferation assay (**l**), colony formation assay (**m**), and trans-well migration assay (**n**) demonstrated that miR-150-5p regulates H1299 cell proliferation and migration. (**o-p**) is the statistical result for (**m-n**). Scale bar = 50μm. **q**-**r** Flow cytometry assay detecting lipid peroxidation levels (**q**) and reactive oxygen species (ROS) levels (**r**) in H1299 cells. Statistical results were also presented. **s** Relative levels of glutathione (GSH), ferrous ions (Fe²^+^), and malondialdehyde (MDA) in H1299 cells after forced expression of miR-150-5p mimics compared with miR-NC group were detected. **t-v** Xenograft tumor formation for H1299 cells treated with miR-150-5p-mimics or miR-NC were examined. Tumor images (**t**), volumes (**u**), and tumor masses (**v**) were presented, respectively. **w-x** Results of immunohistochemical staining(**w**) including quantification(**x**) of positive signals for Ki67, Cleaved Caspase 3 (CC3), and SLC7A11. Scale bar = 50μm. * *P* < 0.05, ** P < 0.01, *** P < 0.001. HPF = high power field.

Based on the classic ceRNA strategy of lncRNA regulating targeted gene expression, the Annolnc^40^, TargetScan^41^, miRDB^42^, and Starbase^43^ databases were collected and analyzed to prognosticate the microRNAs that engage in direct interactions with both SLC7A11AR and SLC7A11. Subsequently, miR-150-5p and miR-142-5p were discerned and designated as the prospective miRNAs involved in such interactions (Fig. 5a). Interestingly, in LUAD, both miR-150-5p and miR-142-5p exhibited a negative correlation with SLC7A11AR (Fig. 5b and Fig. S5a). However, only miR-150-5p, but not miR-142-5p, was identified to be downregulated in LUAD (Fig. 5c-e and Fig S5b-c). Most importantly, the AUC value for miR-150-5p in LUAD was 0.737, analyzed by the ROC curve, indicating that miR-150-5p might function as a prospective independent biomarker for prognostic evaluation (Fig. S5d). Therefore, we conducted a more in-depth characterization of the functional role mediated by miR-150-5p. The results showed that overexpressing miR-150-5p mimics severely reduced SLC7A11AR levels. (Fig. 5f). miR-150-5p was markedly upregulated after SLC7A11AR knockdown (Fig. 5g and Fig. S5e). The miRDB database was used to predict miR-150-5p binding sites on the SLC7A11AR transcript, followed by a dual-luciferase reporter assay for validation (Fig. 5h-i). Concurrently, in H1299, H1975, and SPC-A1 cells, miR-150-5p mimics reduced SLC7A11 expression, while miR-150-5p inhibitors increased its expression (Fig. 5j-k and Fig. S5f-g). Furthermore, miR-150-5p mimics inhibited tumor cell growth and migration, whereas miR-150-5p inhibitors exhibited the controversial effects (Fig. 5l-p and Fig. S5h-l).

**Figure 6:**
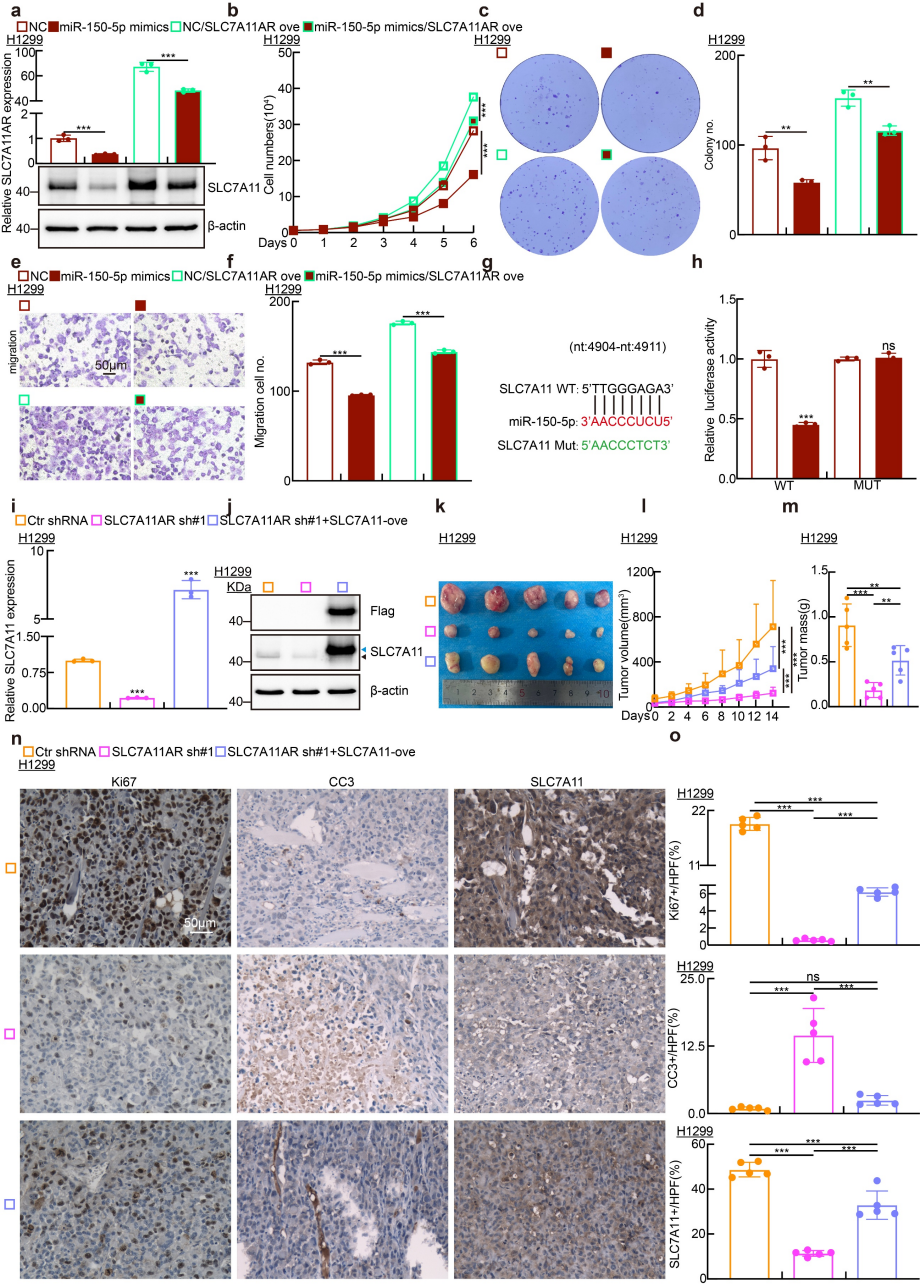
** SLC7A11AR acts as a ceRNA for miR-150-5p to promote SLC7A11 expression in lung adenocarcinoma. a** Relative SLC7A11AR (top) and SLC7A11 protein expressions (bottom) in indicated cells were detected by Real-time RT-PCR and immunoblot, respectively. **b-f** Cell proliferation assay (**b**), colony formation assay (**c-d**), and trans-well migration assay (**e-f**) demonstrated that SLC7A11AR overexpression rescues the inhibitory effects of miR-150-5p mimics, compared with miR-NC group. **g** Predicted binding sites of miR-150-5p to the 3'-UTR region of SLC7A11 and schematic of wild-type (WT) and mutant (MUT) luciferase reporter plasmids for SLC7A11 3'-UTR were shown. **h** HEK-293T cells co-transfected with specific plasmids and miR-NC or miR-150-5p mimics were collected to detect relative luciferase activities. **i-j** Relative RNA (**i**) and protein (**j**) expressions of SLC7A11 in indicated cells were detected by Real-time RT-PCR and immunoblot, respectively. Dark arrow: endogenous SLC7A11 proteins; Blue arrow: exogenous SLC7A11 proteins. **k-m** Forced expression of SLC7A11 can rescue the inhibitory effect induced by adding SLC7A11 shRNA, examined by xenograft tumor formation assay. Tumor images (**k**), tumor volumes (**l**), and tumor weights (**m**) were presented. **n-o** Results of immunohistochemical staining, including quantification of positive signals for Ki67, Cleaved Caspase 3 (CC3), and SLC7A11. Scale bar = 50μm. * *P* < 0.05, ** P < 0.01, *** P < 0.001. HPF = high power field.

In line with the functional role of SLC7A11AR regulating ferroptosis, the levels of GSH in cells significantly decreased, with lipid peroxidation, ROS, MDA, and Fe²⁺ increased upon miR-150-5p mimics overexpression (Fig. 5q-s and Fig. S5m-n). Fer-1 significantly rescued the miR-150-5p mimic-induced suppression of cell growth and migration while markedly reducing the elevated lipid peroxidation level and reactive oxygen species (ROS) caused by miR-150-5p mimics (Fig. S5o-q). As anticipated, miR-150-5p mimics notably suppressed the growth of xenograft tumors (Fig. 5t-x and Fig. S5r-x). The pivotal role of the SLC7A11AR/miR-150-5p/SLC7A11 axis in LUAD was confirmed through growth curve analysis, colony formation, and trans-well assays using reciprocal rescue experiments (Fig. 6a-f and Fig. S6a-l). Subsequently, the 3'-UTR fragment of SLC7A11 was cloned into the pGL3-Basic plasmid, and the transcriptional regulation of SLC7A11 by miR-150-5p was confirmed using a dual-luciferase reporter assay (Fig. 6g-h). Overexpression of SLC7A11 could, to some extent, overcome the cellular effects caused by SLC7A11AR knockdown (Fig. 6i-o). The above results indicated that SLC7A11AR/miR-150-5p/SLC7A11 axis inhibits ferroptosis and promotes LUAD malignant progression.

### Targeting SLC7A11AR with ASO inhibits tumor growth

**Figure 7:**
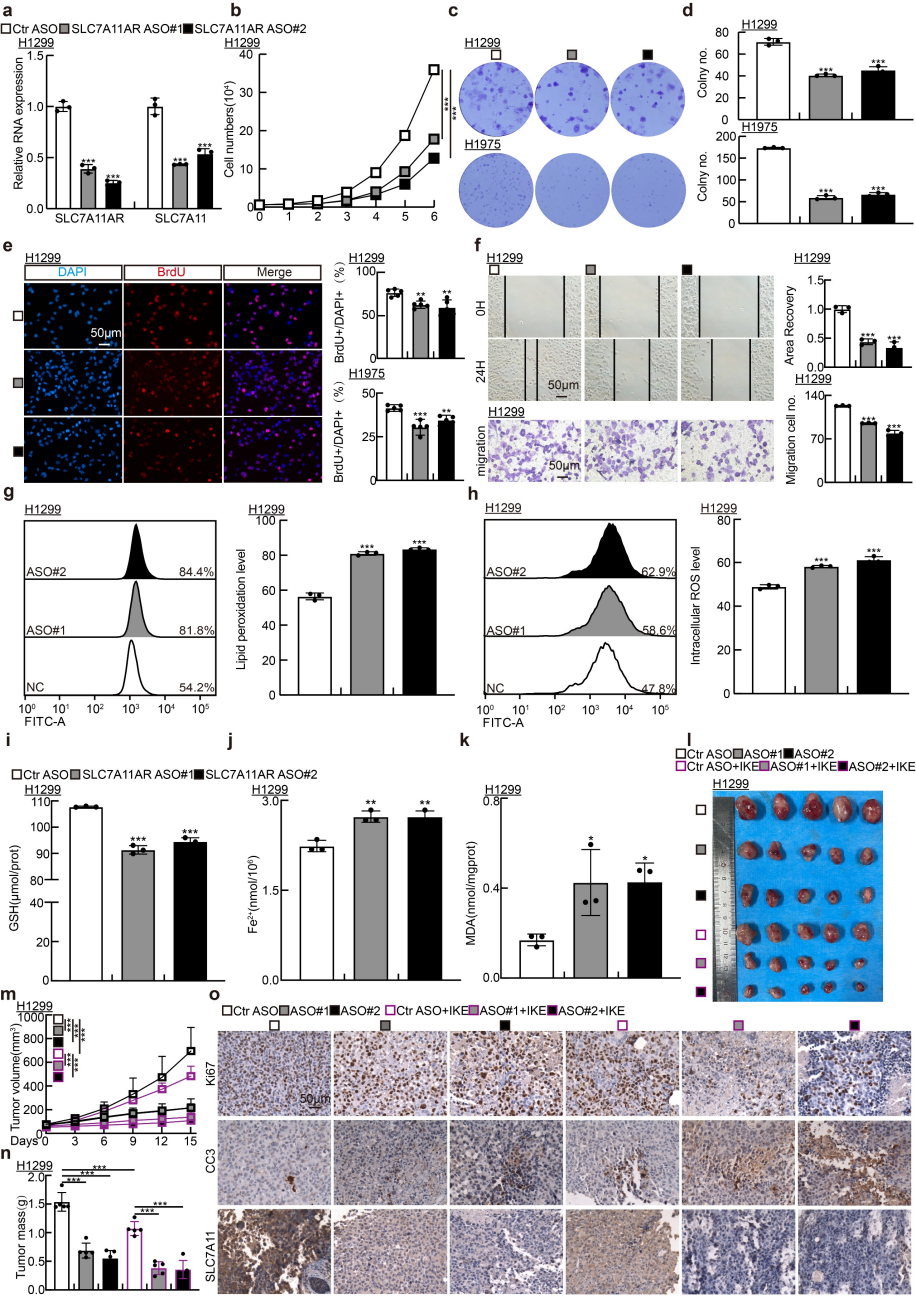
** Targeting SLC7A11AR with specific ASOs repressed tumor growth. a** Relative RNA expression validation of SLC7A11AR and SLC7A11 in H1299 cells post SLC7A11AR-targeting ASO used Real-time RT-PCR. **b-e** Growth curve assay (**b**), colony formation assay (**c-d**), and BrdU incorporation assay (**e**) examined the inhibitory effects of SLC7A11AR-targeting ASOs. Scale bar = 50μm. **f** Wound healing (top) and trans-well migration (bottom) assays were performed to unveil the inhibitory effects of SLC7A11AR-targeting ASOs. Statistical results were presented. Scale bar=50μm. **g-h** Flow cytometry assays were performed to examine the lipid peroxidation levels (**g**) and reactive oxygen species (ROS) levels (**h**) in H1299 cells after SLC7A11AR-targeting ASOs transfection. Statistical results were presented. **i-k** Changes in glutathione (GSH) (**i**), ferrous ions (Fe²⁺)(**j**), and malondialdehyde (MDA) (**k**) levels in H1299 cells after SLC7A11AR-targeting ASO transfection. **l-o** Images of xenograft tumors after SLC7A11AR-targeting ASO treatment, with or without ferroptosis inducer IKE, were examined. Xenograft tumor images (**l**), changes in tumor volume (**m**), and tumor weight (**n**) derived from H1299 cells were presented, respectively. (**o**) Immunohistochemical staining for Ki67, CC3, and SLC7A11 in xenograft tumors. Scale bar = 50μm. *P < 0.05, ** P < 0.01, *** P < 0.001. ASO-NC=ASO control.

Previous investigations have established that antisense oligonucleotide (ASO) medications possess pronounced tumor-suppressive capabilities *in vitro* and *in vivo*^34^. To explore whether ASOs exert comparable inhibitory functions to SLC7A11AR, we designed two specific ASOs targeting different sites on SLC7A11AR, along with a control ASO (Table S2). The indicated ASOs were transfected into H1299, H1975, and SPC-A1 cell lines. The ASOs targeting SLC7A11AR suppressed the expressions of both SLC7A11AR and SLC7A11 in comparison to the control ASO group (Fig. 7a and Fig. S7a). Furthermore, the ASOs targeting SLC7A11AR suppressed the proliferation and migration of tumor cells (Fig. 7b-f and Fig. S7b-f). Flow cytometry results indicated that transfection with ASOs increased intracellular lipid peroxidation and ROS levels (Fig. 7g-h and Fig. S7g-h). In addition, the levels of GSH in the cells significantly decreased, while MDA and Fe²⁺ increased upon SLC7A11AR-targeting ASO treatment (Fig. 7i-k and Fig. S7i-k).

To assess the *in vivo* therapeutic efficacy of ASOs, a xenograft tumor model was utilized, wherein the tumors were treated either with or without the ferroptosis inducer IKE (Fig. 7l). Xenograft tumor volume was grown to approximately 50 mm³, mice were randomly allocated into six groups (ASO-control, SLC7A11AR ASO#1, SLC7A11AR ASO#2, ASO-control plus IKE, SLC7A11AR ASO#1 plus IKE, SLC7A11AR ASO#2 plus IKE). Subsequently, different ASOs were injected around the tumor twice a week, while IKE was delivered intraperitoneally every other day. Compared to the ASO-control group, the SLC7A11AR-targeting ASOs significantly inhibited tumor growth, with even better effects observed in combination with IKE (Fig.7m-n). IHC assay showed a significant decrease in Ki67 and SLC7A11 expression levels, accompanied by increased CC3 and 4-HNE expression (Fig.7o and Fig S7l-p). In addition, we conducted an *in vivo* toxicological assessment. There are no significant changes in body weight, liver morphology, or the enzyme activities of alanine aminotransferase (ALT) and aspartate aminotransferase (AST) in the liver following ASOs/IKE treatment (Fig S7q-t). These results indicate that ASOs will hold great promise for therapeutic applications in LUAD.

## Discussion

Recent findings have shown that controlled inflammation can protect against infections and injuries, but when uncontrolled, it can lead to autoimmune diseases and promote changes that contribute to lung cancer^44^. In cases of excessive tissue damage, the extracellular matrix (ECM) promoting healing may dysregulate, which causes tissue stiffness, necrosis, and a decline in lung function^45^. Furthermore, activated transcription factors and cytotoxic immune cells can heighten tissue inflammation, further complicating the situation^46^. For example, immune cells, like macrophages and neutrophils, secrete cytokines in response to sustained tissue damage. While the inflammatory cytokines can attract more immune cells, they may also promote lung cancer growth by enhancing metabolic changes and creating a favorable environment for tumor development^47^. Inflammations can be triggered by various effects, like chronic infections, exposure to pro-inflammatory chemicals, and abnormal immune responses. For instance, chronic bacterial or viral infections can lead to persistent inflammation that may progress to lung cancer^48^.

Here, we showed that inflammatory signaling-induced lncRNA SLC7A11AR promoted tumor growth via interacting with miR-150-5p, thus increasing SLC7A11 expression and reducing ferroptosis. Although most lncRNAs prefer to be in the nucleus, a subset exhibits activity in the cytoplasm. These cytoplasmic lncRNAs can be translocated to neighboring cells or into the serum via exosome-mediated trafficking^49^. However, miR-150-5p and SLC7A11AR were nearly undetectable in the serum in both healthy individuals and cancer patients (data not shown). These indicated that a more sensitive non-coding RNA detecting system should be applied or developed. These findings suggested that miR-150-5p and SLC7A11AR may serve as potential tumor tissue-based biomarkers rather than blood-based biomarkers. Therefore, it will be interesting to validate whether the tumor microenvironment contributes to miR-150-5p dysregulation by isolating tumor tissue infiltrating immune cells or cancer-associated fibroblasts and examining their functional roles in the future.

Ferroptosis can be triggered and restrained in various human cancers depending on the cellular context. When induced in NSCLC, ferroptosis exhibits a significant inhibitory effect on tumor progression within both *in vitro* and *in vivo* experimental assays^50^-^52^. The primary mechanism behind this effect revolves around the classic GSH-dependent signaling pathways rather than through other pathways that may influence reactive oxygen species (ROS) or cellular iron levels^53^. Remarkably, the tumor suppressor BRCA1-associated protein 1 (BAP1) can inhibit SLC7A11 expression, whose suppression results in lipid peroxidation and facilitates the process of ferroptosis^54^. In certain cancers, Kelch-like ECH-associated protein 1 (KEAP1) reduces SLC7A11 protein stability, thereby impairing the exchange of glutamate and cystine^55^. Conversely, NFE2-related factor 2 (NRF2) enhances SLC7A11 transcription and mitigates KEAP1-mediated degradation, ultimately inhibiting ferroptosis^55^, ^56^. In lung cancer, the RNA-binding protein RBMS1 augmenting the expression of SLC7A11 leads to an elevation in the production of GSH and consequently suppresses ferroptosis^57^. In addition, ADP-ribosylation factor (ARF) inhibits Nrf2 expression and reduces SLC7A11 expression, sensitizing tumor cells to ferroptosis^58^. However, recent contrary findings uncovered that increased SLC7A11 expression unexpectedly induced the sensitivity of tumor cells to oxidative stress, resulting in elevated rates of tumor cell apoptosis^59^.

**Figure 8:**
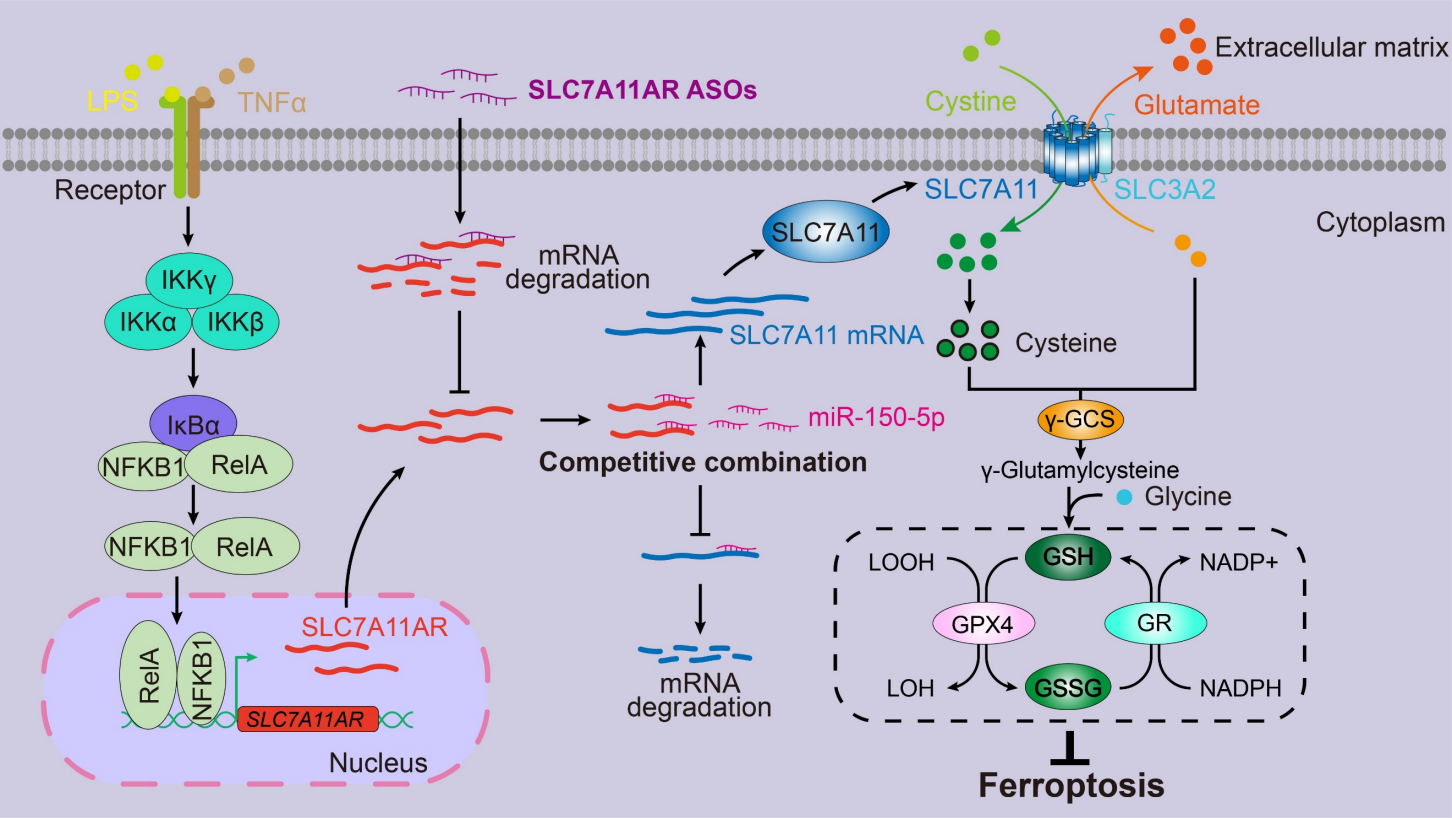
** Proposed model for LncRNA SLC7A11AR promoting LUAD progression via SLC7A11AR/miR-150-5p/SLC7A11 axis**.

Thus, controlling SLC7A11 expression levels might be more effective in manipulating ferroptosis. Several compounds have been developed by inhibiting SLC7A11, including erastin, IKE, sorafenib, and Sulfasalazine (SAS)^60^. HG106 inhibited SLC7A11 expression and tumor growth, especially in KRAS mutant LUAD^61^. Our data showed that the knockdown of SLC7A11AR by specific targeting ASO with or without ferroptosis agonist repressed tumor growth both *in vitro* and *in vivo* (Fig.8). In addition, it will be of great significance to conduct further investigations into the efficacy of combination therapy through the blockade of SLC7A11 expression in conjunction with immune checkpoint inhibitors^62^.

In conclusion, we identified the key lncRNA SLC7A11AR induced by the inflammatory signaling pathway in NSCLC. We also elucidated the crucial function of the SLC7A11AR/miR-150-5p/SLC7A11 axis in facilitating tumor growth by suppressing ferroptosis, thereby presenting novel diagnostic and prognostic targets for the future treatment of LUAD.

## Supplementary Material

Supplementary figures and tables.

## Abbreviations

NSCLC: Non-small cell lung cancer
SCLC: Small cell lung cancer
LUAD: Lung adenocarcinoma
LUSC: Lung squamous cell carcinoma
SLC7A11AR: Solute carrier family 7 member 11-associated lncRNA
ncRNAs: Non-coding RNAs
lncRNAs: Long non-coding RNAs
PCD: Programmed cell death
SLC7A11: Solute carrier family 7 member 11
SLC3A2: Solute carrier family 3 member 2
GPX4: Glutathione Peroxidase 4
FSP1: Ferroptosis Suppressor Protein 1
FTH1: Ferritin heavy chain 1
GSH: Glutathione
GCL: Glutamate-cysteine ligase
ROS: Reactive oxygen species
FBS: Fetal bovine serum
CHIP: Chromatin Immunoprecipitation assay
IHC: Immunohistochemical staining
TEM: Transmission electron microscopy
MDA: Malondialdehyde
Fe^2+^: Ferrous ion
ROC: Receiver operating characteristic
AUC: The area under the curve
LPS: Lipopolysaccharide
TNF-α: Tumor necrosis factor
NFKB1: Nuclear factor kappa B subunit 1 or p50;NFKB2: Nuclear factor kappa B subunit 2
RELA: p65
FISH: Fluorescence in situ hybridization
CC3: Cleaved caspase 3
EMT: Epithelial-mesenchymal transition
ceRNA: Competing endogenous RNA
ASO: Antisense oligonucleotide
ALT: Alanine Aminotransferase
AST: Aspartate Aminotransferase

## References

[1] Bray F, Laversanne M, Sung H, Ferlay J, Siegel RL, Soerjomataram I (2024). Global cancer statistics 2022: GLOBOCAN estimates of incidence and mortality worldwide for 36 cancers in 185 countries. CA Cancer J Clin.

[2] Nicholson AG, Tsao MS, Beasley MB, Borczuk AC, Brambilla E, Cooper WA (2022). The 2021 WHO Classification of Lung Tumors: Impact of Advances Since 2015. J Thorac Oncol.

[3] Sung H, Ferlay J, Siegel RL, Laversanne M, Soerjomataram I, Jemal A (2021). Global Cancer Statistics 2020: GLOBOCAN Estimates of Incidence and Mortality Worldwide for 36 Cancers in 185 Countries. CA Cancer J Clin.

[4] Hanahan D (2022). Hallmarks of Cancer: New Dimensions. Cancer Discov.

[5] Yan H, Bu P (2021). Non-coding RNA in cancer. Essays Biochem.

[6] Hu Q, Ma H, Chen H, Zhang Z, Xue Q (2022). LncRNA in tumorigenesis of non-small-cell lung cancer: From bench to bedside. Cell Death Discov.

[7] Braga EA, Fridman MV, Burdennyy AM, Loginov VI, Dmitriev AA, Pronina IV (2023). Various LncRNA Mechanisms in Gene Regulation Involving miRNAs or RNA-Binding Proteins in Non-Small-Cell Lung Cancer: Main Signaling Pathways and Networks. Int J Mol Sci.

[8] Bach DH, Lee SK (2018). Long noncoding RNAs in cancer cells. Cancer Lett.

[9] Wei MM, Zhou GB (2016). Long Non-coding RNAs and Their Roles in Non-small-cell Lung Cancer. Genomics Proteomics Bioinformatics.

[10] Zhang L, Liang J, Qin H, Lv Y, Liu X, Li Z (2023). Lnc AC016727.1/BACH1/HIF-1 alpha signal loop promotes the progression of non-small cell lung cancer. J Exp Clin Cancer Res.

[11] Jiang Y, Guo H, Tong T, Xie F, Qin X, Wang X (2022). lncRNA lnc-POP1-1 upregulated by VN1R5 promotes cisplatin resistance in head and neck squamous cell carcinoma through interaction with MCM5. Mol Ther.

[12] Chi Y, Wang D, Wang J, Yu W, Yang J (2019). Long Non-Coding RNA in the Pathogenesis of Cancers. Cells.

[13] Sun Y, Peng ZL (2009). Programmed cell death and cancer. Postgrad Med J.

[14] Zou J, Wang L, Tang H, Liu X, Peng F, Peng C (2021). Ferroptosis in Non-Small Cell Lung Cancer: Progression and Therapeutic Potential on It. Int J Mol Sci.

[15] Jiang X, Stockwell BR, Conrad M (2021). Ferroptosis: mechanisms, biology and role in disease. Nat Rev Mol Cell Biol.

[16] Lei G, Zhuang L, Gan B (2022). Targeting ferroptosis as a vulnerability in cancer. Nat Rev Cancer.

[17] Koppula P, Lei G, Zhang Y, Yan Y, Mao C, Kondiparthi L (2022). A targetable CoQ-FSP1 axis drives ferroptosis- and radiation-resistance in KEAP1 inactive lung cancers. Nat Commun.

[18] Liu J, Xia X, Huang P (2020). xCT: A Critical Molecule That Links Cancer Metabolism to Redox Signaling. Mol Ther.

[19] Wang L, Liu Y, Du T, Yang H, Lei L, Guo M (2020). ATF3 promotes erastin-induced ferroptosis by suppressing system Xc(). Cell Death Differ.

[20] Liu MR, Zhu WT, Pei DS (2021). System Xc(-): a key regulatory target of ferroptosis in cancer. Invest New Drugs.

[21] Chen X, Kang R, Kroemer G, Tang D (2021). Broadening horizons: the role of ferroptosis in cancer. Nat Rev Clin Oncol.

[22] Zhang X, Zheng X, Ying X, Xie W, Yin Y, Wang X (2023). CEBPG suppresses ferroptosis through transcriptional control of SLC7A11 in ovarian cancer. J Transl Med.

[23] Zhu H, Xu S (2024). SOX4 inhibits ferroptosis and promotes proliferation of endometrial cancer cells via the p53/SLC7A11 signaling. J Obstet Gynaecol Res.

[24] Singh N, Baby D, Rajguru JP, Patil PB, Thakkannavar SS, Pujari VB (2019). Inflammation and cancer. Ann Afr Med.

[25] Sohrab SS, Raj R, Nagar A, Hawthorne S, Paiva-Santos AC, Kamal MA (2023). Chronic Inflammation's Transformation to Cancer: A Nanotherapeutic Paradigm. Molecules.

[26] Landskron G, De la Fuente M, Thuwajit P, Thuwajit C, Hermoso MA (2014). Chronic inflammation and cytokines in the tumor microenvironment. J Immunol Res.

[27] Tuli HS, Sak K, Iqubal A, Garg VK, Varol M, Sharma U (2022). STAT signaling as a target for intervention: from cancer inflammation and angiogenesis to non-coding RNAs modulation. Mol Biol Rep.

[28] Xiong Q, Jiang L, Liu K, Jiang X, Liu B, Shi Y (2021). miR-133b targets NCAPH to promote beta-catenin degradation and reduce cancer stem cell maintenance in non-small cell lung cancer. Signal Transduct Target Ther.

[29] Tian F, Wang J, Zhang Z, Yang J (2020). LncRNA SNHG7/miR-34a-5p/SYVN1 axis plays a vital role in proliferation, apoptosis and autophagy in osteoarthritis. Biol Res.

[30] Li G, Ma L, He S, Luo R, Wang B, Zhang W (2022). WTAP-mediated m(6)A modification of lncRNA NORAD promotes intervertebral disc degeneration. Nat Commun.

[31] Zhu Y, Zhou B, Hu X, Ying S, Zhou Q, Xu W (2022). LncRNA LINC00942 promotes chemoresistance in gastric cancer by suppressing MSI2 degradation to enhance c-Myc mRNA stability. Clin Transl Med.

[32] Deng Z, Ou H, Ren F, Guan Y, Huan Y, Cai H (2020). LncRNA SNHG14 promotes OGD/R-induced neuron injury by inducing excessive mitophagy via miR-182-5p/BINP3 axis in HT22 mouse hippocampal neuronal cells. Biol Res.

[33] Giridharan S, Srinivasan M (2018). Mechanisms of NF-kappaB p65 and strategies for therapeutic manipulation. J Inflamm Res.

[34] Jiang X, Zhang Y, Yuan Y, Jin Z, Zhai H, Liu B (2023). LncRNA GSCAR promotes glioma stem cell maintenance via stabilizing SOX2 expression. Int J Biol Sci.

[35] Miotto G, Rossetto M, Di Paolo ML, Orian L, Venerando R, Roveri A (2020). Insight into the mechanism of ferroptosis inhibition by ferrostatin-1. Redox Biol.

[36] Dong Y, Wu Y, Zhao GL, Ye ZY, Xing CG, Yang XD (2019). Inhibition of autophagy by 3-MA promotes hypoxia-induced apoptosis in human colorectal cancer cells. Eur Rev Med Pharmacol Sci.

[37] Chang H, Sun F, Tian K, Wang W, Zhou K, Zha D (2020). Caspase inhibitor z-VAD-FMK increases the survival of hair cells after Actinomycin-D-induced damage in vitro. Neurosci Lett.

[38] Li Y, Zeng X, Lu D, Yin M, Shan M, Gao Y (2021). Erastin induces ferroptosis via ferroportin-mediated iron accumulation in endometriosis. Hum Reprod.

[39] Bai Q, Pan Z, Nabi G, Rashid F, Liu Y, Khan S (2022). Emerging role of competing endogenous RNA and associated noncoding RNAs in thyroid cancer. Am J Cancer Res.

[40] Yang DC, Ke L, Ding Y, Gao G (2021). AnnoLnc: A One-Stop Portal to Systematically Annotate Novel Human Long Noncoding RNAs. Methods Mol Biol.

[41] McGeary SE, Lin KS, Shi CY, Pham TM, Bisaria N, Kelley GM (2019). The biochemical basis of microRNA targeting efficacy. Science.

[42] Chen Y, Wang X (2020). miRDB: an online database for prediction of functional microRNA targets. Nucleic Acids Res.

[43] Li JH, Liu S, Zhou H, Qu LH, Yang JH (2014). starBase v2.0: decoding miRNA-ceRNA, miRNA-ncRNA and protein-RNA interaction networks from large-scale CLIP-Seq data. Nucleic Acids Res.

[44] Chen L, Deng H, Cui H, Fang J, Zuo Z, Deng J (2018). Inflammatory responses and inflammation-associated diseases in organs. Oncotarget.

[45] Herrera J, Henke CA, Bitterman PB (2018). Extracellular matrix as a driver of progressive fibrosis. J Clin Invest.

[46] Liaskou E, Wilson DV, Oo YH (2012). Innate immune cells in liver inflammation. Mediators Inflamm.

[47] Kartikasari AER, Huertas CS, Mitchell A, Plebanski M (2021). Tumor-Induced Inflammatory Cytokines and the Emerging Diagnostic Devices for Cancer Detection and Prognosis. Front Oncol.

[48] Wu B, Sodji QH, Oyelere AK (2022). Inflammation, Fibrosis and Cancer: Mechanisms, Therapeutic Options and Challenges. Cancers (Basel).

[49] Qu L, Ding J, Chen C, Wu ZJ, Liu B, Gao Y (2016). Exosome-Transmitted lncARSR Promotes Sunitinib Resistance in Renal Cancer by Acting as a Competing Endogenous RNA. Cancer Cell.

[50] Du L, Yang H, Ren Y, Ding Y, Xu Y, Zi X (2023). Inhibition of LSD1 induces ferroptosis through the ATF4-xCT pathway and shows enhanced anti-tumor effects with ferroptosis inducers in NSCLC. Cell Death Dis.

[51] Wang Z, Shen N, Wang Z, Yu L, Yang S, Wang Y (2024). TRIM3 facilitates ferroptosis in non-small cell lung cancer through promoting SLC7A11/xCT K11-linked ubiquitination and degradation. Cell Death Differ.

[52] Feng Y, Xu J, Shi M, Liu R, Zhao L, Chen X (2022). COX7A1 enhances the sensitivity of human NSCLC cells to cystine deprivation-induced ferroptosis via regulating mitochondrial metabolism. Cell Death Dis.

[53] Yadav P, Sharma P, Sundaram S, Venkatraman G, Bera AK, Karunagaran D (2021). SLC7A11/ xCT is a target of miR-5096 and its restoration partially rescues miR-5096-mediated ferroptosis and anti-tumor effects in human breast cancer cells. Cancer Lett.

[54] Zhang Y, Zhuang L, Gan B (2019). BAP1 suppresses tumor development by inducing ferroptosis upon SLC7A11 repression. Mol Cell Oncol.

[55] Habib E, Linher-Melville K, Lin HX, Singh G (2015). Expression of xCT and activity of system xc(-) are regulated by NRF2 in human breast cancer cells in response to oxidative stress. Redox Biol.

[56] Chang K, Chen Y, Zhang X, Zhang W, Xu N, Zeng B (2023). DPP9 Stabilizes NRF2 to Suppress Ferroptosis and Induce Sorafenib Resistance in Clear Cell Renal Cell Carcinoma. Cancer Res.

[57] Zhang W, Sun Y, Bai L, Zhi L, Yang Y, Zhao Q (2021). RBMS1 regulates lung cancer ferroptosis through translational control of SLC7A11. J Clin Invest.

[58] Chen D, Tavana O, Chu B, Erber L, Chen Y, Baer R (2017). NRF2 Is a Major Target of ARF in p53-Independent Tumor Suppression. Mol Cell.

[59] Jiang Y, Sun M (2024). SLC7A11: the Achilles heel of tumor?. Front Immunol.

[60] Xie Y, Hou W, Song X, Yu Y, Huang J, Sun X (2016). Ferroptosis: process and function. Cell Death Differ.

[61] Hu K, Li K, Lv J, Feng J, Chen J, Wu H (2020). Suppression of the SLC7A11/glutathione axis causes synthetic lethality in KRAS-mutant lung adenocarcinoma. J Clin Invest.

[62] Gao W, Wang X, Zhou Y, Wang X, Yu Y (2022). Autophagy, ferroptosis, pyroptosis, and necroptosis in tumor immunotherapy. Signal Transduct Target Ther.

