# Supplementary material for: LncRNA SLC7A11AR promotes lung adenocarcinoma progression by inhibiting ferroptosis via promoting SLC7A11 expression
Source: Int J Biol Sci. 2025 Jul 11;21(10):4549–66. doi: 10.7150/ijbs.112233 (PMC12320243; doi:10.7150/ijbs.112233)
Supplement: Supplementary file 1 — Supplementary figures and tables. [file ijbsv21p4549s1.pdf]

LncRNA SLC7A11AR promotes lung adenocarcinoma progression by inhibiting ferroptosis via promoting SLC7A11 expression

Haoqing Zhai[1,2,‡], Xudong Xiang[3,‡], Jun Pu[3,‡], Xiaoqun Niu[3,‡], Jie Gao[4], Dengcai Mu[1,2], Jia Du[1,2], Yao Li[1,2], Laihao Qu[4], Baiyang Liu[4,*], Yongbin Chen[1,2,4,*], Cuiping Yang[5,6,*]

1. State Key Laboratory of Genetic Evolution & Animal Models, the key laboratory of Animal Models & Human Disease Mechanisms of Yunnan Province, Kunming Institute of Zoology, Chinese Academy of Sciences, 650201, Kunming, Yunnan, China.
2. Kunming College of Life Science, University of Chinese Academy of Sciences, Beijing, 100049, China.
3. Kunming Medical University, Kunming, Yunnan 650118, China.
4. The First Affiliated Hospital of Zhengzhou University, Zhengzhou 450052, China.
5. The International Peace Maternity and Child Health Hospital, School of Medicine, Shanghai Jiao Tong University, Shanghai, China.
6. Shanghai Key Laboratory of Embryo Original Diseases, Shanghai 200030, China.

‡ These authors contributed equally to this work.

[*] Correspondence email: Cuiping Yang: cuipingyang@sjtu.edu.cn
Yongbin Chen: ybchen@mail.kiz.ac.cn
Baiyang Liu: zzuliubaiyang@163.com

Running title

SLC7A11AR inhibits ferroptosis in lung adenocarcinoma

Conflict of interest

The authors declare no conflict of interest.

Supplementary Information

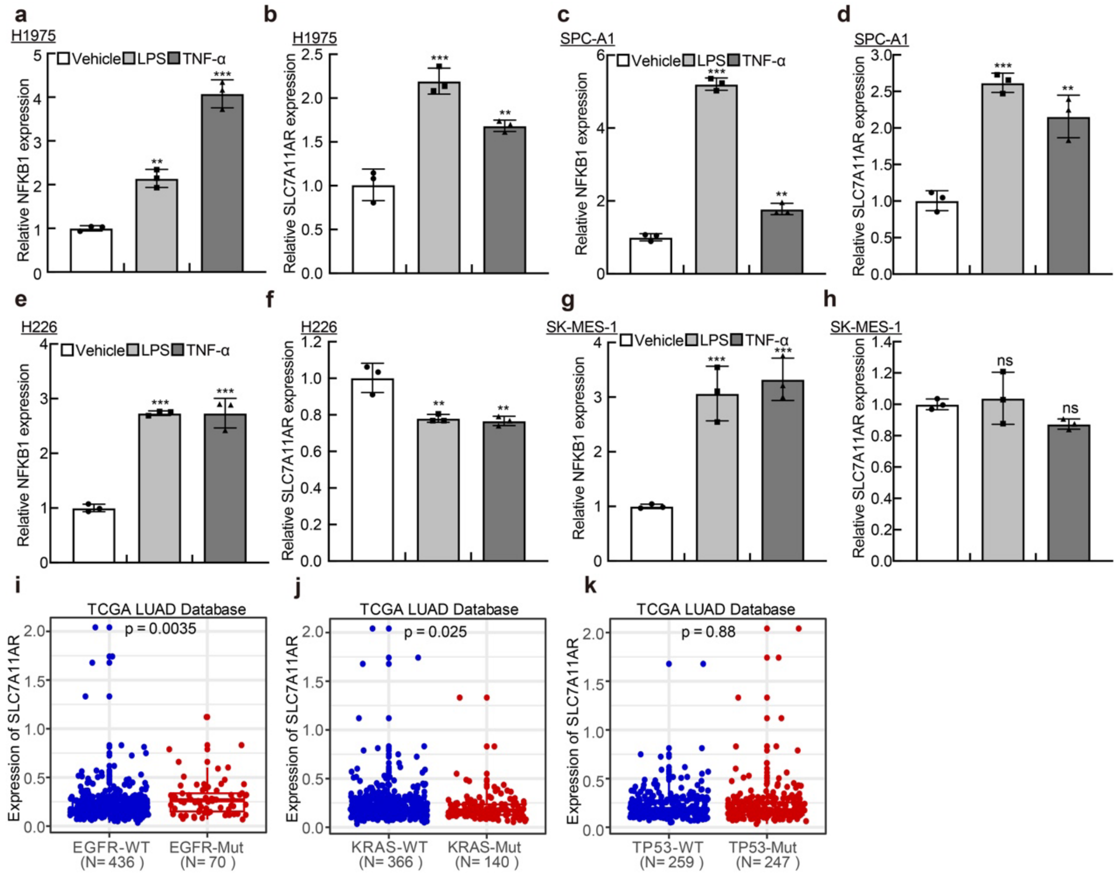

Fig S1. Inflammatory induces lncRNA SLC7A11AR expression in LUAD. a-d Using NFKB1 as a positive control, changes in SLC7A11AR expression in H1975 and SPC-A1 cells after treatment with LPS and TNF-α were examined by Real-time RT-PCR. **e-h** NFKB1 was used as a positive control, and changes in SLC7A11AR expression in LUSC cell lines, including H226 and SK-MES-1, after LPS and TNF-α treatment were examined by Real-time RT-PCR. **i-k** Using the TCGA-LUAD dataset to analyze the correlation between EGFR, KRAS, and TP53 gene mutations and SLC7A11 expression levels. * $P < 0.05$, ** $P < 0.01$, *** $P < 0.001$.

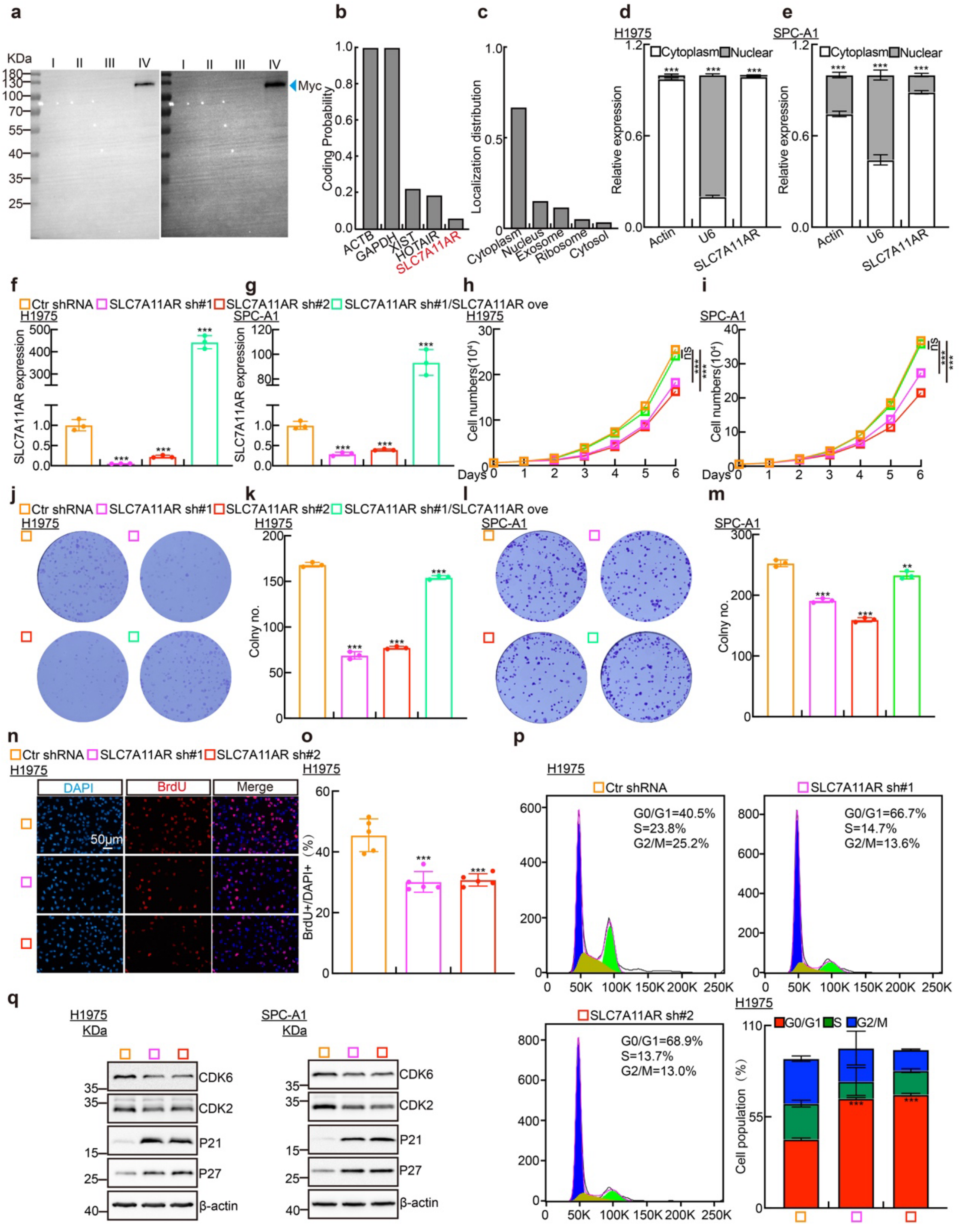

Fig S2. SLC7A11AR promotes LUAD tumor cell proliferation. a An IP to detect whether SLC7A11AR can code for proteins or short peptides. NCAPH-Myc protein was used as a control (blue arrow). The weakly exposed result (left) and the strongly exposed result (right) (I: SLC7A11AR-Myc, II: SLC7A11AR-T-Myc, III: SLC7A11AR-TT-Myc, IV: NCAPH-Myc). **b** Prediction of the protein-coding potential of SLC7A11AR by CPAT. **c** Prediction of subcellular localization of SLC7A11AR by LncLocater. **d-e** Subcellular structural localization of SLC7A11AR detected by

nuclear-cytoplasmic separation experiment (cell fractionation) and Real-time RT-PCR in H1975 (**d**) and SPC-A1 (**e**), respectively. **f-g** Efficiency of SLC7A11AR knockdown and overexpression in H1975 (**f**) and SPC-A1 (**g**) cells assessed by Real-time RT-PCR. **h-o** Knockdown of SLC7A11AR significantly inhibits the proliferation (**h-i**), colony formation (**j-m**), and BrdU incorporation (**n-o**) ability of H1975 and SPC-A1 cells. Reciprocal statistical results were presented. Scale bar = 50μm. **p** PI staining and flow cytometry to assess the impact of SLC7A11AR knockdown on the G0/G1 cell cycle transition in H1975 cells, including quantitative data. **q** Immunoblot to detect the protein expressions of the key cell cycle regulators, including CDK2, CDK6, P21, and P27, following SLC7A11AR knockdown. * $P < 0.05$, ** $P < 0.01$, *** $P < 0.001$. Ove = over-expression; sh#1 = shRNA#1; sh#2 = shRNA#2.

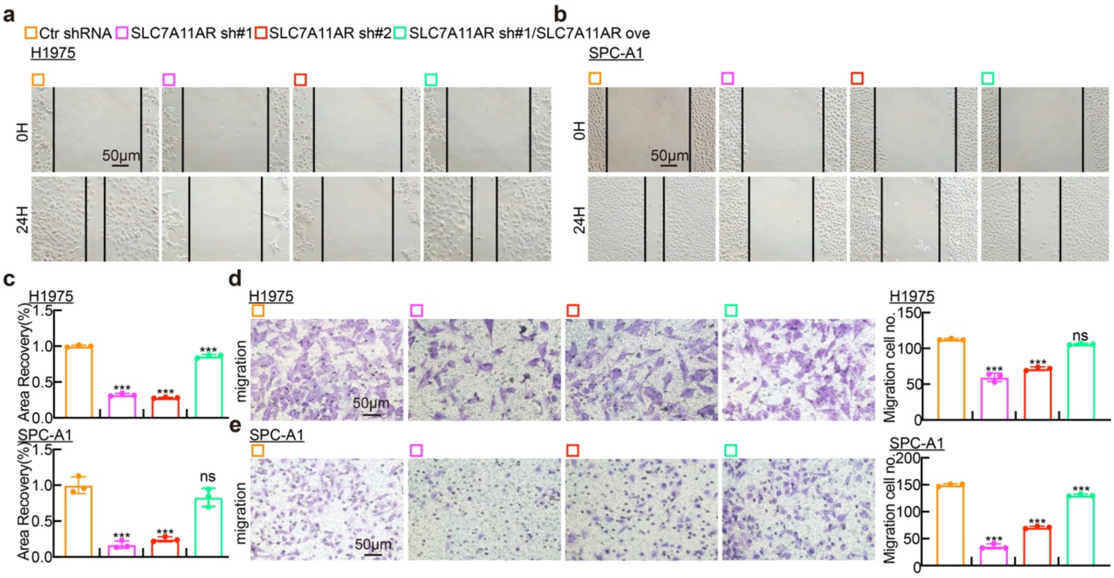

Fig S3. SLC7A11AR plays as an oncogene in LUAD. a-e Wound healing (**a-c**) and trans-well (**d-e**) migration assays in H1975 and SPC-A1 cells following SLC7A11AR knockdown and overexpression were performed, with quantitative statistics presented. Scale bar = 50μm. * $P < 0.05$, ** $P < 0.01$, *** $P < 0.001$. Ove= over-expression; sh#1 = shRNA#1; sh#2 = shRNA#2.

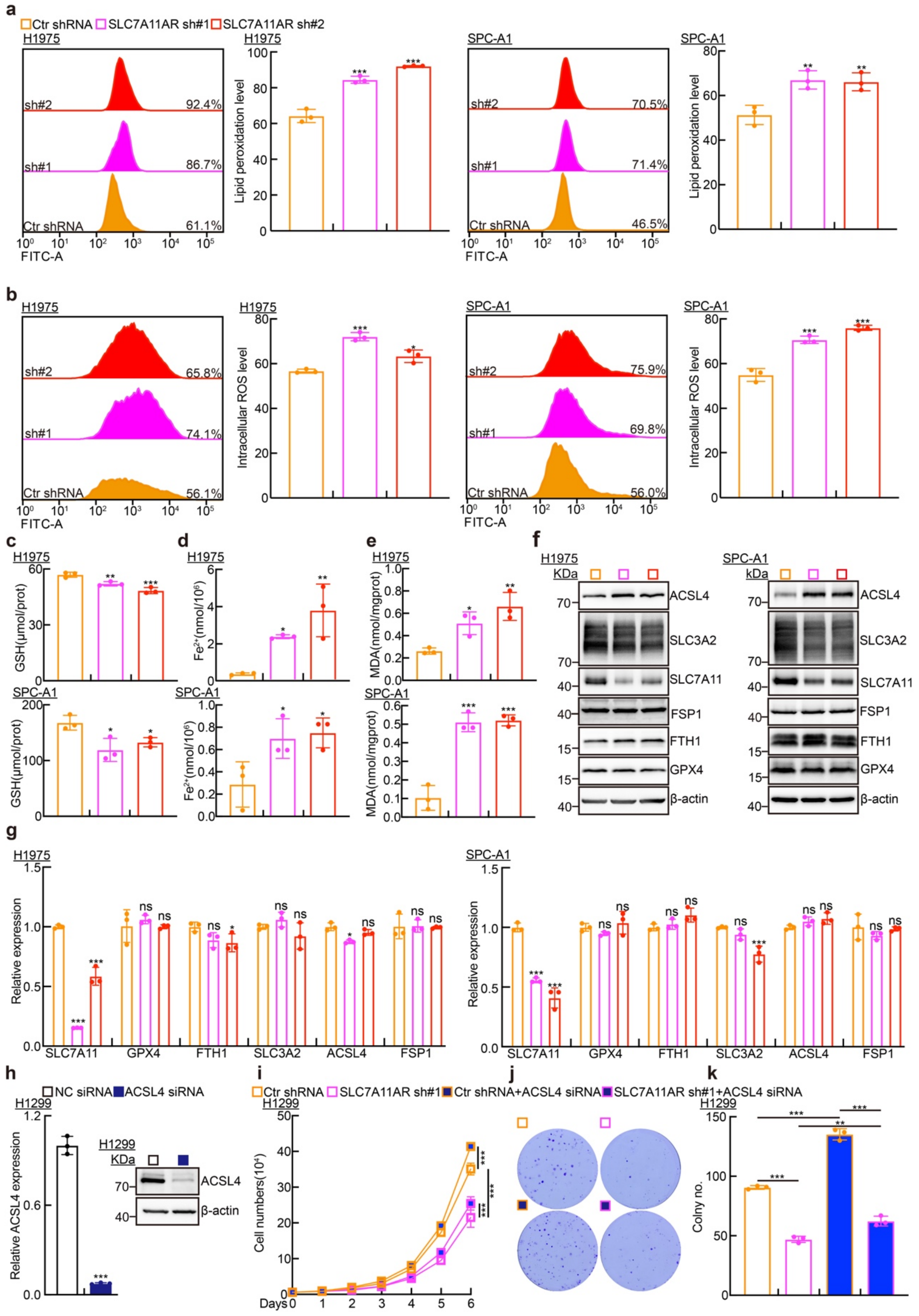

Fig S4. SLC7A11AR promotes SLC7A11 expression at the transcriptional level and inhibits ferroptosis. a-b Flow cytometry detecting lipid peroxidation (**a**) and intracellular reactive oxygen species (ROS) levels (**b**) in H1975 and SPC-A1 cells after SLC7A11AR knockdown, including statistical results for both. **c-e** Detection of

glutathione (GSH) **(c)**, ferrous ions (Fe^{2+}) **(d)**, and malondialdehyde (MDA) **(e)** levels in H1975 and SPC-A1 cells, respectively, after SLC7A11AR knockdown. **f-g** Immunoblot and Real-time RT-PCR assays to detect protein (**f**) and the RNA (**g**) expressions of the key regulators in ferroptosis signaling following SLC7A11AR knockdown in indicated cells. **h** Validation of ACSL4 knockdown efficiency using targeted siRNAs, including RT-qPCR (left) and immunoblot analysis (right). **i-k** ACSL4 knockdown rescued the growth inhibition **(i)** and colony formation suppression **(j-k)** induced by SLC7A11AR knockdown. * $P < 0.05$, ** $P < 0.01$, *** $P < 0.001$.

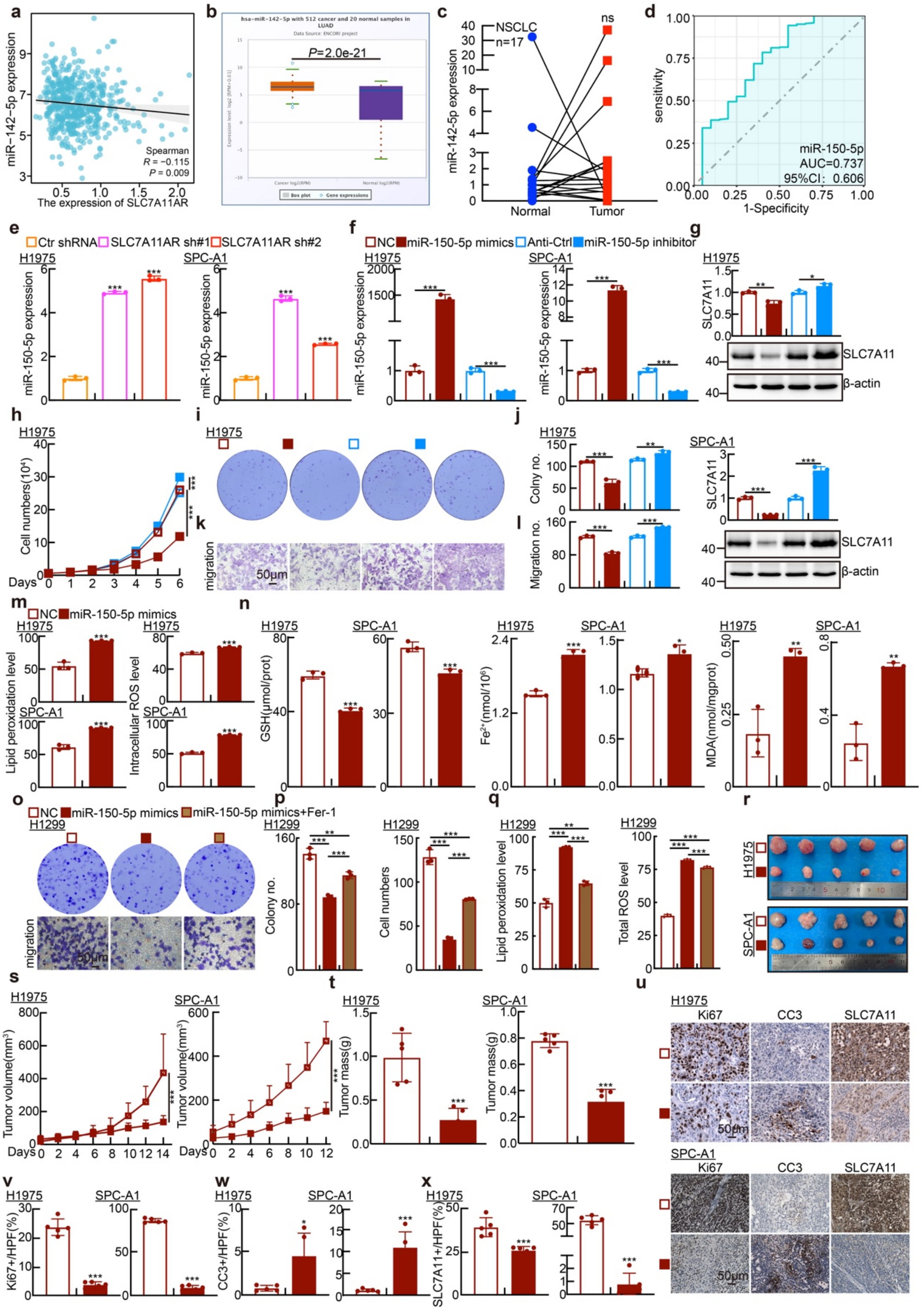

Fig S5. MiR-150-5p promotes ferroptosis in lung adenocarcinoma. **a** Correlation analysis of SLC7A11AR with miR-142-5p using the TCGA-LUAD dataset. **b** Relative expression levels of miR-142-5p in TCGA-LUAD (Normal: 20 cases; Tumor: 512 cases). **c** Detection of expression levels of miR-142-5p in paired clinical tissue samples

from LUAD patients by Real-time RT-PCR (n=17). **d** ROC curve analysis of miR-150-5p in lung adenocarcinoma using the TCGA dataset (AUC=0.737). **e** Relative expressions of miR-150-5p in H1975 and SPC-A1 cells after SLC7A11AR knocking down were measured using Real-time RT-PCR. **f** Transfection efficiency of miR-150-5p mimics and inhibitors in H1975 and SPC-A1 cells. **g** Changes in expression levels of SLC7A11 in H1975 and SPC-A1 cells after transfection with miR-150-5p mimics and inhibitors, assessed by Real-time RT-PCR (top) and immunoblot (bottom). **h-l** Cell proliferation and migration abilities of H1975 cells after transfection with miR-150-5p mimics and inhibitors were evaluated using growth curve assay (**h**), colony formation assay (**i-j**), and trans-well migration assay (**k-l**), including statistical results; Scale bar=50μm. **m** Flow cytometry assay detecting lipid peroxidation levels and reactive oxygen species (ROS) levels in H1975 and SPC-A1 cells demonstrate that miR-150-5p mimics promote ferroptosis. Statistical results were presented. **n** The relative expressions of glutathione (GSH), ferrous ions (Fe^{2+}), and malondialdehyde (MDA) in H1975 and SPC-A1 cells after forced expression of miR-150-5p mimics were detected and compared with miR-NC group. **o-p** Cell proliferation and migration abilities of H1299 cells after transfection with miR-150-5p mimics and after treatment with Fer-1 were evaluated using colony formation assay and trans-well migration assay, including statistical results; Scale bar=50μm. **q** Flow cytometry assay detecting lipid peroxidation levels and reactive oxygen species (ROS) levels in H1299 cells demonstrates that Fer-1 inhibits miR-150-5p-mediated ferroptosis. Statistical results were presented. **r-t** Images of xenograft tumors formed by H1975 and SPC-A1 cells treated with miR-150-5p-mimics or miR-NC (**r**), changes in tumor volumes (**s**), and tumor weights (**t**) were presented, respectively. **u-x** Results of immunohistochemical staining (**u**), including quantification of positive signals for Ki67, Cleaved Caspase 3 (CC3), and SLC7A11 (**v-x**) were shown. Scale bar=50μm. * $P < 0.05$, ** $P < 0.01$, *** $P < 0.001$. HPF = high power field.

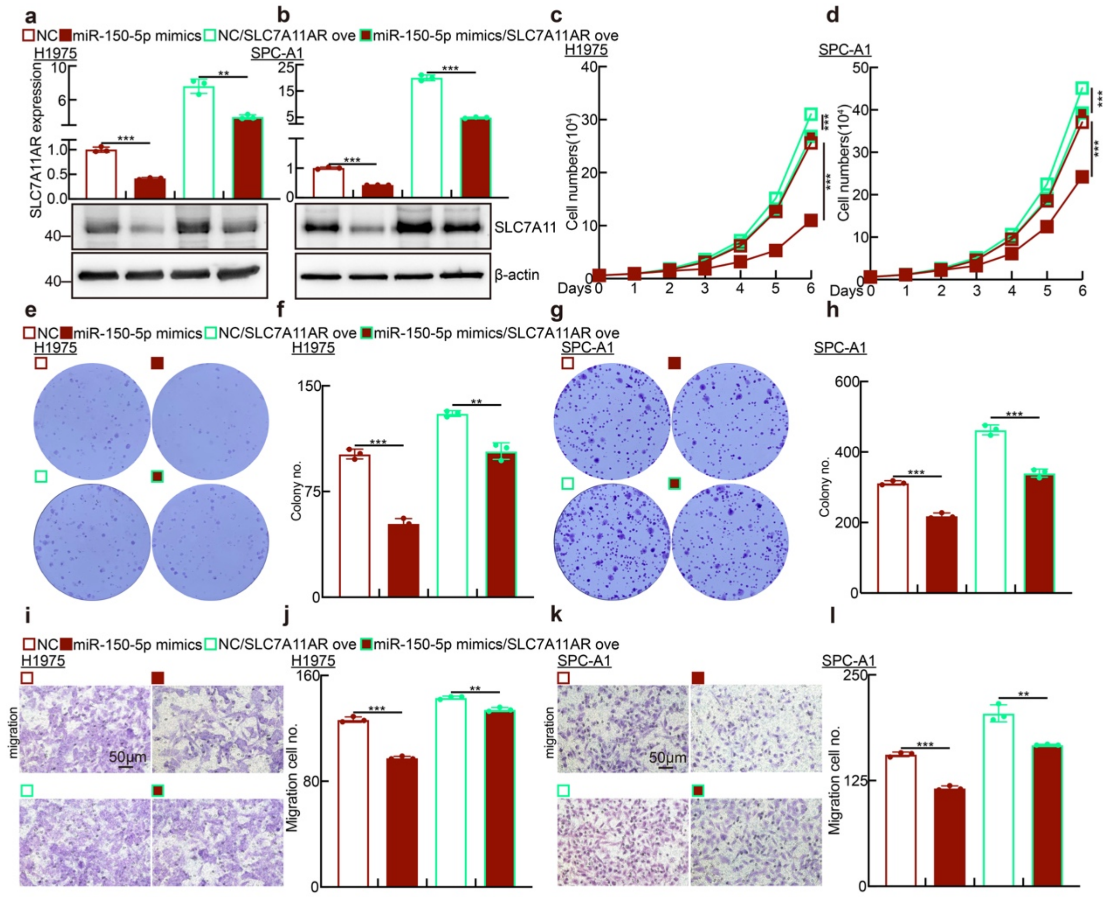

Fig S6. SLC7A11AR acts as a ceRNA for miR-150-5p to promote SLC7A11 expression in lung adenocarcinoma. a-b Relative SLC7A11AR RNA (top) and protein (bottom) expressions of SLC7A11 in indicated cells were detected by Real-time RT-PCR and immunoblot, respectively. **c-l** Cell proliferation assays (**c-d**), colony formation assays (**e-h**), and trans-well migration assays (**i-l**) were performed to show that SLC7A11AR overexpression rescues the inhibitory effects of miR-150-5p mimics. Statistical results were presented. * $P < 0.05$, ** $P < 0.01$, *** $P < 0.001$.

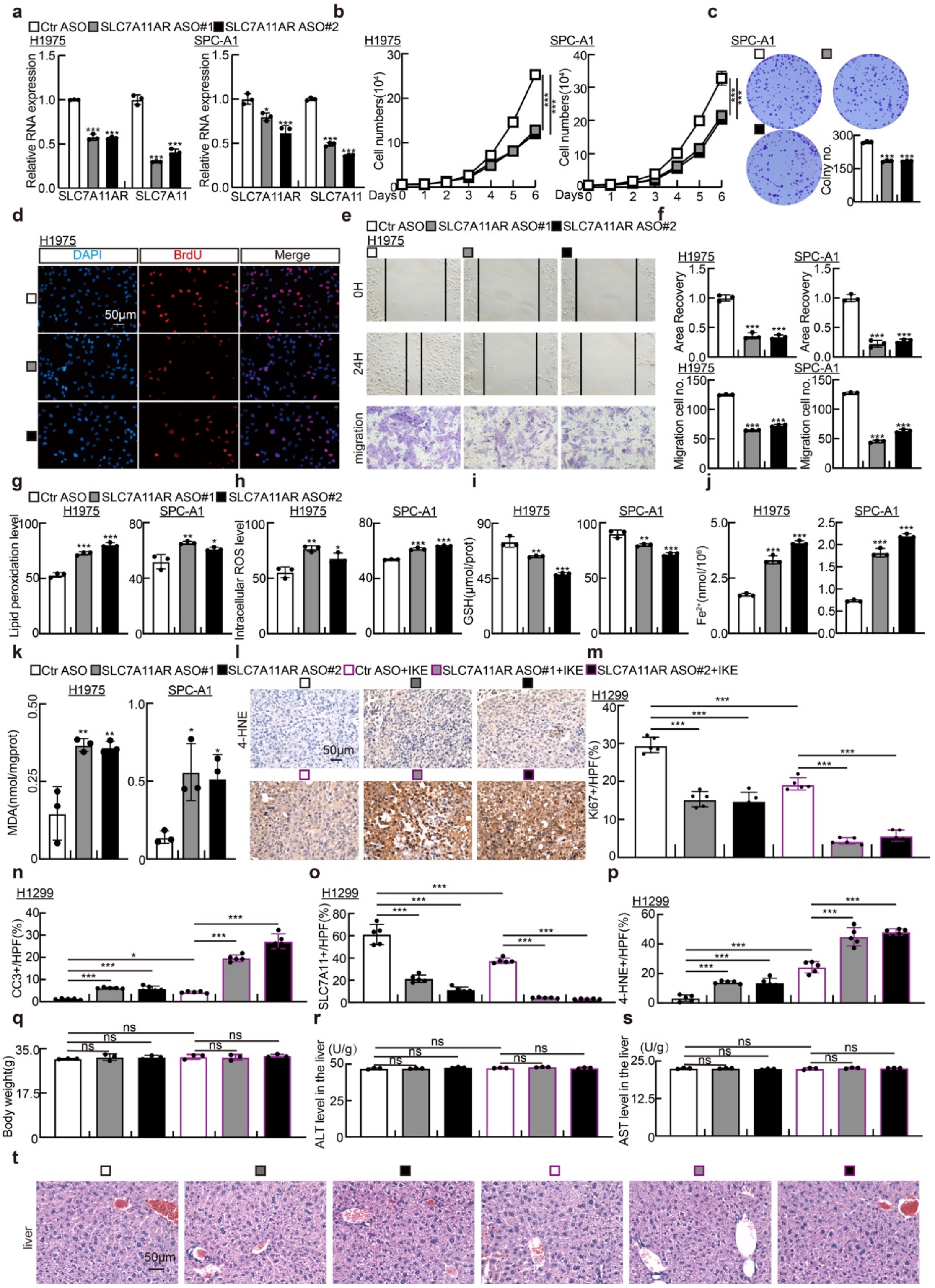

Fig S7. Targeting SLC7A11AR with specific ASOs retarded tumor growth. a The relative RNA expressions of SLC7A11AR and SLC7A11 in H1975 and SPC-A1 cells after transfection with SLC7A11AR-targeting ASOs were examined by Real-time RT-PCR. **b-d** Growth curve assays (**b**), colony formation assays (**c**), and BrdU incorporation assays (**d**) demonstrating the proliferation inhibitory effects of

SLC7A11AR-targeting ASOs in indicated cells were presented. Scale bar = 50μm. **e-f** Wound healing and trans-well migration assays indicating the migration inhibitory effects of SLC7A11AR-targeting ASOs in indicated cells with statistical data included. Scale bar =50μm. **g-h** Flow cytometry analysis detecting lipid peroxidation levels and reactive oxygen species (ROS) levels in H1975 and SPC-A1 cells after SLC7A11AR-targeting ASOs transfection, including statistical results for both assays. **i-k** Changes in the levels of glutathione (GSH) (**i**), ferrous ions (Fe^{2+}) (**j**), and malondialdehyde (MDA) (**k**) in H1975 and SPC-A1 cells following SLC7A11AR-targeting ASOs transfection. (**l**) Immunohistochemical staining for 4-HNE in xenograft. **m-p** quantification of the positive signals for Ki67 (**m**), Cleaved Caspase 3 (CC3) (**n**), SLC7A11 (**o**) and 4-HNE(**p**). Scale bar = 50μm. **q** Statistical results of mouse body weight. **r-t** ALT(**r**) and AST(**s**) activity in mouse liver was measured, and H&E staining (**t**) in liver. * $P < 0.05$, ** $P < 0.01$, *** $P < 0.001$.

Table 1. The candidate lncRNAs were identified by integrative analyses using various GEO datasets.

GSE81089	GSE144520	GSE1541
AL390786.1	AC092447.4	AC008080.1
AC034187.1	AC008771.1	AC020659.1
AC009690.2	AL161629.1	DIRC1
AC003005.2	AP003465.1	SNHG11
AC104561.3	RORA-AS1	AC008771.1
AC008771.1	SNHG12	MIR1-1HG
AC078860.2	AP002856.2	TUG1
AC099788.1	AC004832.6	SNHG29
TUG1	LINC01851	TMEM51-AS1
AC025871.2	LINC00942	LINC00942
BET1-AS1	AC003005.2	AC007601.1
AC073525.1	LINC01686	LINC00476
AC009269.3	AC099506.1	LINC00152
AC138207.4	LINC01091	C15orf54
AC073655.1	LINC00152	LINC01106
LINC00942	AL354928.1	ZBTB44-DT
LUARIS	LINC02686	LINC02694
LINC00504	AC114498.1	LINC02880
AC078922.1	LINC01734	DLEU1
AC027237.2	TMEM72-AS1	SNHG14
AL590378.1	SNHG14	AL138899.1
SNHG14	LINC01531	AC104129.1
LINC01256	ZFAND2A-DT	AL033381.1
NORAD	AC080013.3	NORAD
WAC-AS1	NORAD	C14orf177
AL133371.2	SNHG7	C20orf197
LINC02740	logFC>3, p<0.01	TTTY14
ITCH-AS1		C15orf56
AL355300.1		LINC00303
DIRC1		MIR7-3HG
LINC02365		AL358781.1
CREB3L2-AS1		TYMSOS
LINC01792		AP000679.1

LINC01227		IRAG1-AS1
AATBC		PRDM16-DT
AC011374.1		LINC01561
AC069277.1		C8orf31
AL049874.3		DLGAP1-AS1
DUXAP8		LINC00469
PRKAG2-AS1		FLJ13224
SNHG7		FLJ40194
AL109947.1		SNHG7
AC073127.1		ZFAS1
LINC01679		C1orf167-AS1
AC104063.1		AL355390.1
AC079349.1		CASC2
AC012349.1		AC011944.1
AC069133.1		AC105206.1
TMSB15B-AS1		AC025171.1
AL138831.1		FAM87B
JPX		AL162595.1
LINC01340		TMEM78
AP000786.1		TENM3-AS1
AL596188.1		ZNRF3-AS1
FP325332.1		AL161668.1
LINC01031		FAM27E5
AC138331.1		C9orf62
AC007879.3		FAM230I
LINC00917		AC068473.1
LINC01620		LINC00314
AL512625.1		C5orf64
TPT1-AS1		LMO7DN

LINC00208		ADORA2A-AS1
SHANK2-AS3		LINC00324
MIR31HG		AC245177.1
C11orf40		AC020907.1
PRSS30P		C9orf106
MIR4435-2HG		LINC00670
DELEC1		MTUS2-AS1
CCDC13-AS1		LINC00311
AC008080.1		AL162457.1
AC020659.1		LINC00174
DIRC1		IL6-AS1
SNHG11		SLC24A3-AS1
MIR1-1HG-AS1		LINC01699
MIR1-1HG		EIF3J-DT
SNHG10		AP003471.1
logFC>3, p<0.01		LINC00305
		FLJ37453
		AC135776.1
		PCBP1-AS1
		PIK3CD-AS1
		LINC00652
		LINC02870
		ACTA2-AS1
		PRNT
		LINC00304
		AC022148.1
		PRR26
		C9orf139
		LINC02363

		WDFY3-AS2
		CSNK1G2-AS1
		LINC01559
		LINC01555
		LINC02878
		AC004832.1
		FER1L6-AS1
		HECW1-IT1
		C6orf223
		LINC00471
		CELF2-AS1
		LINC02724
		OGFRP1
		TP53TG1
		PRR34
		C17orf77
		FAM87A
		AC138028.1
		LINC00334
		LINC01006
		AC239585.1
		FAM230E
		PRKCZ-AS1
		TSPEAR-AS2
		PRORY
		AC138356.1
		WT1-AS
		LINC01547
		AC005037.1

		logFC>3, p<0.01

AC00877.1: SLC7A11AR

GSE81089: Next Generation Sequencing (RNAseq) from NSCLC.

GSE144520: whole-transcriptome sequencing of A549 cells and cisplatin-resistant A549/DPP cells.

GSE1541: whole-transcriptome sequencing of A549 cells and A549 treated with inflammatory factors (LPS and TNF-α).

Table 2. Antibodies and oligos used in this study.

Antibody Name	Catalog number	Dilution	Supplier	Species
NFKB1（p50）	13586	1:50（CHIP）	CST	Rabbit
IgG	2729	1:500（CHIP）	CST	Rabbit
Myc	2278	1:2000	CST	Rabbit
CDK2	10122-1-AP	1:2000	Proteintech	Rabbit
CDK6	124821	1:2000	Proteintech	Rabbit
P21	2947	1:1000	CST	Rabbit
P27	610241	1:2000	BD	Mouse
β-actin	60008-1-1g	1:5000	Proteintech	Mouse
E-cadherin	3195	1:2000	CST	Rabbit
N-cadherin	ab18203	1:1000	abcam	Rabbit
Vimentin	103661-1-AP	1:2000	Proteintech	Rabbit
Flag	14793	1:2000	CST	Rabbit
ACSL4	sc-271800	1:1000	Santa Cruz	Mouse
SLC3A2	47213	1:1000	CST	Rabbit
SLC7A11	ab307601	1:500	abcam	Rabbit
FTH1	4393	1:1000	CST	Rabbit
GPX4	52455	1:1000	CST	Rabbit
FSP1	20886-1-AP	1:1000	Proteintech	Rabbit
4-HNE	ab48506	1:50	abcam	Mouse
**Oligo name**	**Primer sequences（5’-3’）**			
SLC7A11AR_F	GTTGAAGTGTGAGGCGTGAA			

SLC7A11AR_R	TTTCACCATGTTGGTCAGGA
18sRNA_F	GTAACCCGTTGAACCCCATT
18sRNA_R	CCATCCAATCGGTAGTAGCG
NFKB1（P50）_F	TCCATATTTGGGAAGGCCTGAAC
NFKB1（P50）_R	ATGGGCCATCTGTTGGCAG
CHIP#1_F	AGCTTTGTCCCAGATTTGTTTGG
CHIP#1_R	AAGATCTGTGCTAAACCTCCGT
CHIP#2_F	TGGGATAGTATAAGATGCAGG
CHIP#2_R	ACAGGCACCACTACTAACTTT
CHIP#3_F	AAAGTTAGTAGTGGTGCCTGT
CHIP#3_R	CCAGGTCCTGATAAATGCCAT
CHIP#4_F	AGGAACCTACTGACCCAGAC
CHIP#4_R	CGTTAGGGGAGGTCGAGAAT
Human-Actin_F	GACCTGACTGACTACCTCATGAAGAT
Human-Actin_R	GTCACACTTCATGATGGAGTTGAAGG
Human-U6-qPCR	CCAAGCTTCACCCATTCCTAACAGGAC
Human-SLC7A11_F	ATGCAGTGGCAGTGACCTTT
Human-SLC7A11_R	GGCAACAAAGATCGGAACTG
Human-GPX4_F	GCTCCATGCACGAGTTTTCC

Human-GPX4_R	GCTAGAAATAGTGGGGCAGGT
Human-FTH1_F	AAGCTGCAGAACCAACGAGG
Human-FTH1_R	AGTCACACAAATGGGGGTCATT
Human-SLC3A2_F	CTGGTGCCGTGGTCATAATC
Human-SLC3A2_R	GCTCAGGTAATCGAGACGCC
Human-ACSL4_F	GCTACTTGCCTTTGGCTCATGTGC
Human-ACSL4_R	GTGTGGGCTTCAGTACAGTACAGTCTCC
Human-FSP_F	GACTCCTTCCACCACAATGTGG
Human-FSP_R	CAGCACCATCTGGTTCTTCAGG
hsa-miR-142-5p_qPCR	CATAAAGTAGAAAGCACTACT
hsa-miR-150-5p_qPCR	TCTCCCAACCCTTGTACCAGTG
NFKB1 si#1_sense	GGCAGAAGAUGAUCCAUAUTT
NFKB1 si#1_antisense	AUAUGGAUCAUCUUCUGCCTT
NFKB1 si#2_sense	CCCAUACCUUCAAAUAUUATT
NFKB1 si#2_antisense	UAAUAUUUGAAGGUAUGGGTT

ACSL4 siRNA_sense	GCAGAGAUAUCUUGCUUUATT
ACSL4 siRNA_antisense	UAAAGCAAGAUAUCUCUGCTT
SLC7A11AR-sh#1 Forward oligos	CCGGGCGTGAAAGGGTATGTCTGATCTCGAGATCAGACATACCCTTTCACGC TTTTTG
SLC7A11AR-sh#1 Reverse oligos	AATTCAAAAAGCGTGAAAGGGTATGTCTGATCTCGAGATCAGACATACCCTTTCACGC
SLC7A11AR-sh#2 Forward oligos	CCGGGTGAGGCGTGAAAGGGTATGTCTCGAGACATACCCTTTCACGCCTCACTTTTTG
SLC7A11AR-sh#2 Reverse oligos	AATTCAAAAAGTGAGGCGTGAAAGGGTATGTCTCGAGACATACCCTTTCACGCCTCAC
SLC7A11ARove oligo_F	CGGCTAGCATGGTCAGAAAGCCTGTTGTG
SLC7A11ARove oligo_R	CGGAATTCTAACTTATCTTCTTCTGGTAC
miR-NC	UUGUACUACACAAAAGUACUG
hsa-miR-142-5p mimics_sense	CAUAAAGUAGAAAGCACUACU
hsa-miR-142-5p mimics_antisense	UAGUGCUUUCUACUUUAUGUU

hsa-miR-150-5p mimics_sense	UCUCCCAACCCUUGUACCAGUG
hsa-miR-150-5p mimics_antisense	CUGGUACAAGGGUUGGGAGAUU
Anti-Ctrl	CAGUACUUUUGUGUAGUACAA
hsa-miR-150-5p inhibitor	CACUGGUACAAGGGUUGGGAGA
ASO-NC	GCGUATTATAGCCGATTAAC
SLC7A11AR ASO#1	TGTTGAAATTCGTGCTCCAC
SLC7A11AR ASO#2	AGTGATGGCAGATTTCTCAT

Table 3. The predicted SLC7A11AR downstream targeted miRNAs examined by Annolnc, Starbase, Mirdb and TargetScan, respectively.

Annolnc	Starbase-1	Mirdb	TargetScan	Starbase-2
hsa-miR-802	hsa-miR-545-5p	hsa-miR-1297	hsa-miR-142-5p	hsa-let-7a-5p
hsa-miR-142-5p	hsa-miR-212-3p	hsa-miR-26a-5p	hsa-miR-150-5p	hsa-let-7b-5p
hsa-miR-146-5p	hsa-miR-132-3p	hsa-miR-548c-3p	hsa-miR-144-3p	hsa-let-7c-5p
hsa-miR-192-5p	hsa-miR-1179	hsa-miR-26b-5p	hsa-miR-27a-3p	hsa-let-7d-5p
hsa-miR-215-5p	hsa-miR-545-3p	hsa-miR-3163	hsa-miR-27b-3p	hsa-let-7e-5p
hsa-miR-217	hsa-miR-3622a-5p	hsa-miR-8485	hsa-miR-3681-3p	hsa-let-7f-5p
hsa-miR-30-5p	hsa-miR-520h	hsa-miR-5011-5p	hsa-miR-128-3p	hsa-miR-17-5p
hsa-miR-338-3p	hsa-miR-520g-3p	hsa-miR-4465	hsa-miR-216a-3p	hsa-miR-142-5p
hsa-miR-9-5p	hsa-miR-589-5p	hsa-miR-190a-3p	hsa-miR-375	hsa-miR-18a-5p
hsa-miR-1-3p	hsa-miR-2115-3p	hsa-miR-513a-3p	hsa-miR-384	hsa-miR-19a-3p
hsa-miR-206	hsa-miR-340-5p	hsa-miR-651-3p	hsa-miR-532-5p	hsa-miR-19b-3p
hsa-miR-132-3p	hsa-miR-5590-3p	hsa-miR-4495	hsa-miR-142-3p.2	hsa-miR-20a-5p
hsa-miR-212-3p	hsa-miR-142-5p	hsa-miR-513c-3p	hsa-miR-26a-5p	hsa-miR-21-5p
hsa-miR-133a-3p.2	hsa-miR-519d-3p	hsa-miR-590-3p	hsa-miR-26b-5p	hsa-miR-23a-3p
hsa-miR-133b	hsa-miR-526b-3p	hsa-miR-3662	hsa-miR-1297	hsa-miR-24-3p
hsa-miR-140-3p.2	hsa-miR-20a-5p	hsa-miR-302c-5p	hsa-miR-4465	hsa-miR-25-3p
hsa-miR-141-3p	hsa-miR-93-5p	hsa-miR-3606-3p	hsa-miR-30c-5p	hsa-miR-26a-5p
hsa-miR-200a-3p	hsa-miR-106b-5p	hsa-miR-6867-5p	hsa-miR-30b-5p	hsa-miR-26b-5p
hsa-miR-143-3p	hsa-miR-20b-5p	hsa-miR-9985	hsa-miR-30a-5p	hsa-miR-27a-3p
hsa-miR-150-5p	hsa-miR-17-5p	hsa-miR-27a-3p	hsa-miR-30d-5p	hsa-miR-28-5p

hsa-miR-17-5p	hsa-miR-106a-5p	hsa-miR-27b-3p	hsa-miR-30e-5p	hsa-miR-150-5p
hsa-miR-20-5p	hsa-miR-302e	hsa-miR-144-3p	hsa-miR-452-5p	hsa-miR-30a-5p
hsa-miR-93-5p	hsa-miR-520b	hsa-miR-452-5p	hsa-miR-892c-3p	hsa-miR-31-5p
hsa-miR-106-5p	hsa-miR-520c-3p	hsa-miR-4262	hsa-miR-4676-3p	hsa-miR-32-5p
hsa-miR-519-3p	hsa-miR-520e	hsa-miR-5003-3p	hsa-miR-194-5p	hsa-miR-33a-5p
hsa-miR-183-5p.1	hsa-miR-520a-3p	hsa-miR-4676-3p	hsa-miR-142-3p.1	hsa-miR-92a-3p
hsa-miR-183-5p.2	hsa-miR-520d-3p	hsa-miR-892c-3p	hsa-miR-199b-3p	hsa-miR-93-5p
hsa-miR-200bc-3p	hsa-miR-302a-3p	hsa-miR-33a-3p	hsa-miR-199a-3p	hsa-miR-95-3p
hsa-miR-429	hsa-miR-302b-3p	hsa-miR-5571-5p	hsa-miR-3129-5p	hsa-miR-96-5p
hsa-miR-203a-3p.2	hsa-miR-302c-3p	hsa-miR-378a-5p	hsa-miR-4262	hsa-miR-98-5p
hsa-miR-204-5p	hsa-miR-302d-3p	hsa-miR-369-3p	hsa-miR-181c-5p	hsa-miR-101-3p
has-miR-211-5p	hsa-miR-372-3p	hsa-miR-543	hsa-miR-181b-5p	hsa-miR-105-5p
hsa-miR-223-3p	hsa-miR-373-3p	hsa-miR-577	hsa-miR-181d-5p	hsa-miR-106a-5p
hsa-miR-23-3p	hsa-miR-520f-3p	hsa-miR-5582-3p	hsa-miR-181a-5p	hsa-miR-192-5p
hsa-miR-302-3p	hsa-miR-1323	hsa-miR-1200	hsa-miR-431-5p	hsa-miR-196a-5p
hsa-miR-372-3p	hsa-miR-548o-3p	hsa-miR-340-5p	hsa-miR-148b-3p	hsa-miR-197-3p
hsa-miR-373-3p	hsa-miR-505-3p	hsa-miR-7159-5p	hsa-miR-148a-3p	hsa-miR-199a-5p
hsa-miR-520-3p	hsa-miR-5586-5p	hsa-miR-181c-5p	hsa-miR-152-3p	hsa-miR-199a-3p
hsa-miR-302c-3p.2	hsa-miR-545-3p	hsa-miR-181d-5p	hsa-miR-143-3p	hsa-miR-208a-3p
hsa-miR-425-5p	hsa-miR-1277-5p	hsa-miR-3143	hsa-miR-6088	hsa-miR-129-5p
hsa-miR-7-5p	hsa-miR-3617-5p	hsa-miR-181a-5p	hsa-miR-4770	hsa-miR-148a-3p

	hsa-miR-641	hsa-miR-216b-3p	hsa-miR-5590-3p	hsa-miR-30c-5p
	hsa-miR-671-3p	hsa-miR-548x-3p	hsa-miR-4262	hsa-miR-30d-5p
	hsa-miR-498	hsa-miR-181b-5p	hsa-miR-181c-5p	hsa-miR-139-5p
	hsa-miR-2114-3p	hsa-miR-128-3p	hsa-miR-181b-5p	hsa-miR-147a
	hsa-miR-6823-3p	hsa-miR-548aj-3p	hsa-miR-181d-5p	hsa-miR-7-5p
	hsa-miR-520a-5p	hsa-miR-216a-3p	hsa-miR-181a-5p	hsa-miR-10a-5p
	hsa-miR-525-5p	hsa-miR-6126	hsa-miR-1-3p	hsa-miR-10b-5p
	hsa-miR-552-3p	hsa-miR-32-5p	hsa-miR-206	hsa-miR-181a-5p
	hsa-miR-1193	hsa-miR-548ae-3p	hsa-miR-613	hsa-miR-181b-5p
	hsa-miR-371a-5p	hsa-miR-548aq-3p	hsa-miR-376a-3p	hsa-miR-181c-5p
	hsa-miR-150-5p	hsa-miR-92b-3p	hsa-miR-376b-3p	hsa-miR-182-5p
	hsa-miR-520h	hsa-miR-551b-5p	hsa-miR-363-3p	hsa-miR-183-5p
	hsa-miR-520g-3p	hsa-miR-548j-3p	hsa-miR-25-3p	hsa-miR-199b-5p
	hsa-miR-106b-5p	hsa-miR-587	hsa-miR-32-5p	hsa-miR-205-5p
	hsa-miR-526b-3p	hsa-miR-1305	hsa-miR-92b-3p	hsa-miR-210-3p
	hsa-miR-519d-3p	hsa-miR-6833-3p	hsa-miR-367-3p	hsa-miR-212-3p
	hsa-miR-17-5p	hsa-miR-4668-3p	hsa-miR-92a-3p	hsa-miR-215-5p
	hsa-miR-20a-5p	hsa-miR-3168	hsa-miR-489-3p	hsa-miR-216a-5p
	hsa-miR-93-5p	hsa-miR-92a-3p	hsa-miR-26a-5p	hsa-miR-217
	hsa-miR-106a-5p	hsa-miR-4282	hsa-miR-26b-5p	hsa-miR-218-5p
	hsa-miR-20b-5p	hsa-miR-4999-3p	hsa-miR-1297	hsa-miR-221-3p
	hsa-miR-302e	hsa-miR-548ah-3p	hsa-miR-4465	hsa-miR-222-3p

	hsa-miR-520e	hsa-miR-548am-3p	hsa-miR-4480	hsa-miR-223-3p
	hsa-miR-520b	hsa-miR-605-5p	hsa-miR-192-3p	hsa-miR-224-5p
	hsa-miR-302d-3p	hsa-miR-513a-5p	hsa-miR-6721-5p	hsa-miR-200b-3p
	hsa-miR-372-3p	hsa-miR-410-3p	hsa-miR-635	hsa-let-7g-5p
	hsa-miR-520c-3p	hsa-miR-3065-5p	hsa-miR-6774-5p	hsa-let-7i-5p
	hsa-miR-520a-3p	hsa-miR-4789-3p	hsa-miR-3189-3p	hsa-miR-1-3p
	hsa-miR-520d-3p	hsa-miR-4803	hsa-miR-3127-3p	hsa-miR-23b-3p
	hsa-miR-302a-3p	hsa-miR-367-3p	hsa-miR-6756-3p	hsa-miR-27b-3p
	hsa-miR-302b-3p	hsa-miR-4517	hsa-miR-4711-3p	hsa-miR-30b-5p
	hsa-miR-302c-3p	hsa-miR-363-3p	hsa-miR-592	hsa-miR-122-5p
	hsa-miR-373-3p	hsa-miR-7c-3p	hsa-miR-5010-3p	hsa-miR-125b-5p
	hsa-miR-512-3p	hsa-miR-6873-3p	hsa-miR-2113	hsa-miR-128-3p
		hsa-miR-4768-5p	hsa-miR-6883-3p	hsa-miR-130a-3p
		hsa-miR-3681-3p	hsa-miR-454-5p	hsa-miR-132-3p
		hsa-miR-25-3p	hsa-miR-605-5p	hsa-miR-135a-5p
		hsa-miR-7152-5p	hsa-miR-4668-3p	hsa-miR-137
		hsa-let-7f-2-3p	hsa-miR-548c-3p	hsa-miR-138-5p
		hsa-miR-4775	hsa-miR-548ao-5p	hsa-miR-141-3p
		hsa-miR-1185-1-3p	hsa-miR-548ax	hsa-miR-142-3p
		hsa-miR-3074-5p	hsa-miR-5585-5p	hsa-miR-143-3p
		hsa-miR-494-3p	hsa-miR-3646	hsa-miR-144-3p
		hsa-miR-1185-2-3p	hsa-miR-3662	hsa-miR-152-3p

		hsa-miR-576-5p	hsa-miR-3942-3p	hsa-miR-153-3p
		hsa-miR-142-3p	hsa-miR-4305	hsa-miR-9-5p
		hsa-miR-12120	hsa-miR-517-5p	hsa-miR-125a-5p
		hsa-miR-607	hsa-miR-1251-5p	hsa-miR-149-5p
		hsa-miR-12122	hsa-miR-4684-5p	hsa-miR-154-3p
		hsa-miR-12136	hsa-miR-5571-5p	hsa-miR-185-5p
		hsa-miR-3148	hsa-miR-2054	hsa-miR-186-5p
		hsa-miR-3606-5p	hsa-miR-382-5p	hsa-miR-188-5p
		hsa-miR-150-5p	hsa-miR-374a-3p	hsa-miR-190a-5p
		hsa-miR-487a-5p	hsa-miR-345-5p	hsa-miR-193a-3p
		hsa-miR-487b-5p	hsa-miR-548c-3p	hsa-miR-194-5p
		hsa-miR-101-3p	hsa-miR-1277-5p	hsa-miR-206
		hsa-miR-568	hsa-miR-3146	hsa-miR-320a
		hsa-miR-1277-5p	hsa-miR-5680	hsa-miR-200c-3p
		hsa-miR-335-3p	hsa-miR-3606-3p	hsa-miR-155-5p
		hsa-miR-5590-3p	hsa-miR-513a-3p	hsa-miR-106b-5p
		hsa-miR-380-3p	hsa-miR-513c-3p	hsa-miR-200a-3p
		hsa-miR-142-5p	hsa-miR-5583-5p	hsa-miR-302a-3p
		hsa-miR-489-3p	hsa-miR-3129-3p	hsa-miR-299-3p
		hsa-miR-5700	hsa-miR-376c-3p	hsa-miR-301a-3p
		……	……	……

Annolnc: MicroRNA molecules with potential binding ability to SLC7A11AR were screened through Annolnc database.

Starbase-1: MicroRNAs with potential binding ability to SLC7A11AR were screened through Starbase database.
Mirdb： MicroRNAs with potential binding ability to SLC7A11 were screened through Mirdb database.
TargetScan： MicroRNAs with potential binding ability to SLC7A11 were screened through TargetScan database.
Starbase-2: MicroRNAs with potential binding ability to SLC7A11 were screened through Starbase database.

Table 4. The pathological characteristics of patients with non-small cell lung cancer (NSCLC) donors.

Patients characteristics	No. (%)
**NSCLC patients**	
**Age(years)**	
≤50	0
>50	17（100%）
**Gender**	
Male	7（41%）
Female	10（59%）
